# Cerebroplacental ratio in predicting adverse perinatal outcome: a meta‐analysis of individual participant data

**DOI:** 10.1111/1471-0528.16287

**Published:** 2020-06-08

**Authors:** CA Vollgraff Heidweiller‐Schreurs, IR van Osch, MW Heymans, W Ganzevoort, LJ Schoonmade, CJ Bax, BWJ Mol, CJM de Groot, PMM Bossuyt, MA de Boer, the CPR IPD Study Group, Asma Khalil, Basky Thilaganathan, Ozhan M Turan, Sarah Crimmins, Chris Harman, Alisson M Shannon, Sailesh Kumar, Patrick Dicker, Fergal Malone, Elizabeth C Tully, Julia Unterscheider, Isabella Crippa, Alessandro Ghidini, Nadia Roncaglia, Patrizia Vergani, Amarnath Bhide, Francesco D'Antonio, Gianluigi Pilu, Alberto Galindo, Ignacio Herraiz, Alicia Vázquez‐Sarandeses, Cathrine Ebbing, Synnøve L Johnsen, Henriette O Karlsen

**Affiliations:** ^1^ Department of Obstetrics and Gynaecology, Reproduction and Development Amsterdam UMC Vrije Universiteit Amsterdam Amsterdam The Netherlands; ^2^ Department of Epidemiology and Biostatistics Amsterdam UMC Vrije Universiteit Amsterdam Amsterdam The Netherlands; ^3^ Department of Obstetrics and Gynaecology, Reproduction and Development Amsterdam UMC University of Amsterdam Amsterdam The Netherlands; ^4^ Department of Medical Library Amsterdam UMC Vrije Universiteit Amsterdam Amsterdam The Netherlands; ^5^ Department of Obstetrics and Gynaecology Monash University Clayton Vic. Australia; ^6^ Department of Clinical Epidemiology, Biostatistics and Bioinformatics Amsterdam UMC University of Amsterdam Amsterdam The Netherlands

**Keywords:** Cerebroplacental ratio, Doppler, fetal growth restriction, individual participant data, meta‐analysis, middle cerebral artery, prognostic accuracy

## Abstract

**Objective:**

To investigate if cerebroplacental ratio (CPR) adds to the predictive value of umbilical artery pulsatility index (UA PI) alone – standard of practice – for adverse perinatal outcome in singleton pregnancies.

**Design and setting:**

Meta‐analysis based on individual participant data (IPD).

**Population or sample:**

Ten centres provided 17 data sets for 21 661 participants, 18 731 of which could be included. Sample sizes per data set ranged from 207 to 9215 individuals. Patient populations varied from uncomplicated to complicated pregnancies.

**Methods:**

In a collaborative, pooled analysis, we compared the prognostic value of combining CPR with UA PI, versus UA PI only and CPR only, with a one‐stage IPD approach. After multiple imputation of missing values, we used multilevel multivariable logistic regression to develop prediction models. We evaluated the classification performance of all models with receiver operating characteristics analysis. We performed subgroup analyses according to gestational age, birthweight centile and estimated fetal weight centile.

**Main outcome measures:**

Composite adverse perinatal outcome, defined as perinatal death, caesarean section for fetal distress or neonatal unit admission.

**Results:**

Adverse outcomes occurred in 3423 (18%) participants. The model with UA PI alone resulted in an area under the curve (AUC) of 0.775 (95% CI 0.709–0.828) and with CPR alone in an AUC of 0.778 (95% CI 0.715–0.831). Addition of CPR to the UA PI model resulted in an increase in the AUC of 0.003 points (0.778, 95% CI 0.714–0.831). These results were consistent across all subgroups.

**Conclusions:**

Cerebroplacental ratio added no predictive value for adverse perinatal outcome beyond UA PI, when assessing singleton pregnancies, irrespective of gestational age or fetal size.

**Tweetable abstract:**

Doppler measurement of cerebroplacental ratio in clinical practice has limited added predictive value to umbilical artery alone.

## Tweetable abstract

Doppler measurement of cerebroplacental ratio in clinical practice has limited added predictive value to umbilical artery alone.

## Introduction

Fetoplacental Doppler ultrasound is the most widespread method of fetal monitoring, next to cardiotocography, aiming to predict adverse perinatal outcome.^1^, ^2^ Currently, ultrasonic assessment of the cerebroplacental ratio (CPR) is becoming widely introduced in clinical practice.^3^, ^4^ This test has gained increasing popularity, as shown by the fact that no fewer than six reviews have been published on the subject over the past 3 years.^5^, ^6^, ^7^, ^8^, ^9^, ^10^ It has been ascribed specific potential in detecting late‐onset fetal growth restriction (FGR).^3^, ^5^

The CPR is calculated as the ratio of middle cerebral artery (MCA) to umbilical artery (UA) pulsatility index (PI) values, measured by Doppler ultrasound.^11^ High UA PI values and low MCA PI values are associated with adverse outcomes. As such, CPR has been hypothesised to be more accurate than its individual components.^12^ Multiple clinical trials^1^ have shown UA PI to be a useful surveillance tool in high‐risk pregnancies and UA PI has become standard of practice in FGR pregnancies. The available evidence of CPR and MCA PI is, however, based on a wide range of observational studies, with variable results but most showing an association between low CPR and adverse perinatal outcome.^8^, ^13^ It currently remains unclear whether assessment of CPR adds value to measuring only UA PI and, if so, how well CPR performs in different subpopulations, such as FGR versus normal fetal size.^14^

In our study group’s previous systematic review and meta‐analysis,^8^ predictive value of CPR was comparable to that of UA Doppler measurement for three out of five assessed outcomes and outperformed UA Doppler measurement for the two other outcomes (a composite adverse outcome – differently defined across studies – and emergency delivery for fetal distress). As a consequence, no clear conclusion could be drawn from these results. Furthermore, several factors limited interpretation and subgroup analyses, such as suboptimal reporting of inclusion criteria, large heterogeneity in outcome reporting and the use of different test‐positivity thresholds across studies.^8^ These problems are not uncommon in systematic reviews of prognostic studies (summarised in Panel 1), and could be overcome, in part, by analysing the individual participant data (IPD) collected in these studies.^15^

**Panel 1:** Problems with systematic reviews of published prognostic studies (adapted from Altman 2001^14^)

Difficulty of identifying all studies Negative (non‐significant) results may not be reported (publication bias) Inadequate reporting of methods Variation in study design Most studies are retrospective Variation in inclusion criteria Lack of recognised criteria for quality assessment Different assays or measurement techniques Variation in methods of analysis Differing methods of handling of continuous variables (some dependent on data) Different statistical methods of adjustment Adjustment for different sets of variables Inadequate reporting of quantitative information on outcome Variation in presentation of results (for example, survival at different time‐points)

We here report an IPD meta‐analysis to assess the added value of CPR to the established UA PI as a prognostic antenatal ultrasonic test for adverse perinatal outcome, overall and in subpopulations of women defined by gestational age and fetal size.

## Methods

### Study design and participants

This IPD meta‐analysis was performed according to a prospectively constructed protocol, registered in the PROSPERO database (https://www.crd.york.ac.uk/prospero/display_record.asp?ID=CRD42017072136). The report follows the Preferred Reporting Items for Systematic reviews and Meta‐Analyses (PRISMA) guidelines for meta‐analysis of individual participant data.^16^ Participants were not involved in the development of this study.

During the recruitment stage, we produced a list of potential participating centres by updating the literature search performed for our previously published review of CPR^8^ in PubMed, Embase.com, the Cochrane Library (via Wiley) and ClinicalTrials.gov from inception to 11 July 2017. The full search strategy can be found in the Supplementary material (Appendix S1). No language barriers were used. Studies were eligible that had reported on the association between MCA or CPR indices and perinatal outcome in 200 or more singleton pregnancies without major chromosomal or structural abnormalities diagnosed before birth. We chose beforehand not to include studies with <200 participants, in order to primarily include the larger, generally better performed observational studies. Informed consent was not additionally sought from the study participants, because all obtained data were anonymised.

Subsequently, research groups of eligible reports were contacted and invited to participate in the IPD meta‐analysis and to send the raw data of their study. We identified 55 eligible reports of studies meeting all inclusion criteria (see Supplementary material, Appendix S2). The authors of these reports were contacted. Overall, authors of 25 reports responded positively. Authors of four reports were untraceable; authors of 14 other reports did not respond to several reminders. Authors of 12 reports were unable to share data for various reasons: data were no longer available for five reported studies, authors of one conference abstract preferred to publish their data before sharing the data for this IPD, authors of one report were prevented by strict data‐sharing laws, and authors of another report did not agree to the conditions joining this IPD.

Contributing authors were able to supply the required data from 21 studies within project time limits, consisting of 17 data sets. We received the following information from each contributing centre: anonymised patient identifiers, all baseline demographics and clinical characteristics of participants available (maternal age, hypertensive disorders, other pregnancy complications), gestational age (GA) at delivery, GA at last ultrasound examination before delivery, ultrasonic values of fetal biometry and estimated fetal weight (EFW), ultrasonic values of CPR, MCA PI and UA PI, and perinatal, neonatal and long‐term outcomes available. Birthweight (BW) and EFW centiles were recalculated according to the Intergrowth‐21st standards.^17^, ^18^, ^19^ In case of unknown sex, BW centiles of male and female were averaged. The absolute EFW values could not be recalculated, because the separate biometry measurements were not available in all studies. In line with the most recent American College of Obstetricians and Gynecologists practice bulletin,^20^ the term ‘fetal growth restriction’ was used in this study to describe fetuses with an EFW centile <10, whereas the term small‐for‐gestational age was used to describe newborns with a BW centile <10.

### Quality and risk of bias assessment

Range and consistency checks were performed on the received data sets by two authors (CVHS and IO). Any missing data, obvious errors, inconsistencies between variables or extreme values were queried and rectified as necessary. If details of the study had been published, these were also checked against the raw data and any inconsistencies were similarly queried. All changes made to the data originally supplied by the authors and the reasons for these changes were recorded (see Supplementary material, Appendix S3). Applicability concerns and risk of bias of the individual data sets were assessed independently by two reviewers (CVHS and MB) with the QUADAS‐2 (Quality Assessment of Diagnostic Accuracy Studies‐2) instrument.^21^ In domain 4 (‘Flow and timing’), the time interval between test and delivery was considered not applicable for scoring risk of bias in the prognostic accuracy studies included in this IPD meta‐analysis.

### Outcomes

The main outcome was a composite of adverse perinatal outcome, defined as one or more of the following: perinatal death, emergency caesarean section (CS) for fetal distress and neonatal unit admission. The different data sets employed different definitions of admission to the Neonatal department, and we therefore summarised this variable into any ‘neonatal unit admission’ (referring to admission to the Neonatal intensive care unit (NICU), Neonatal unit or Neonatal critical care unit). Secondary outcomes were the individual components of the main outcome, stillbirth, Apgar score <7 at 5 minutes and acidosis. No core outcome set was used.

### Statistical analysis

In all analyses, a one‐stage IPD approach was used, in which the IPD from all studies were modeled simultaneously while accounting for the clustering of participants within centres by use of a mixed‐effects model. PI values as measured by Doppler ultrasound in the UA and MCA (and subsequently calculation of CPR) were included as continuous variables. In the primary analysis and subgroup analyses of GA and BW centile, multiple imputations were performed on missing data, generating 40 data sets for those variables in which the percentage of missing data did not exceed 40%. In the end, data were missing and imputed in maximally 10.3% of the included cases. Missing data could not be imputed for the subgroup analysis of EFW and the sensitivity analyses, because data were missing for more than 40% of study participants.

Associations between the last measurement before delivery of UA PI, MCA PI and CPR and occurrence of the main outcome were tested using univariate logistic regression. To investigate if CPR improved goodness‐of‐fit, compared with UA PI alone, we added CPR to the model of UA PI using multivariable logistic regression. The discriminative ability of UA PI, MCA PI, CPR and UA PI plus CPR combined was quantified by estimating the areas under the receiver operating characteristics (ROC) curves (AUCs), based on the corresponding model. Multiple imputations were generated using the Multivariate Imputation by Chained Equations (MICE) method^22^ and statistical test results were pooled using Rubin’s Rules for coefficients, standard errors and AUCs (after natural log transformation).^23^

Doppler measurements may differ in prognostic accuracy, depending on the duration of pregnancy and fetal size. We therefore performed subgroup analyses according to GA at delivery, BW centile and EFW centile.

Sensitivity analyses were performed to explore the effect of decisions made within the study process. Sensitivity analyses included the complete‐case data set for the secondary outcomes, time between test and delivery, maternal hypertension, maternal diabetes, a separate subgroup of participants with only CS and a separate subgroup of BW centile <10 at GA ≥34 weeks. As a comparison to the multiple imputation model, we repeated the main analyses in the complete‐case data set. Finally, we repeated the main analysis for addition of MCA PI to UA PI, to test the effect of another combination. Data were managed in IBM SPPS, Version 22.0; analyses were executed in R software (packages lme4).^24^

### Funding

None.

## Results

### Data characteristics

We obtained data of 21 661 individual women from 17 data sets collected in seven countries, after exclusion of 1096 women. Characteristics of the data sets and reasons for exclusion are shown in the Supplementary material (Appendix S4). Eight sets of data (*n* = 4593) had been prospectively collected, and nine data sets (*n* = 17 068) had been retrospectively extracted from patient records. Sample sizes ranged from 207 to 9215. Patient populations varied between the 17 data sets: six (*n* = 2437) were growth restricted or small‐for‐gestational age (varying definitions), six (*n* = 4988) were uncomplicated, four (*n* = 14 243) were mixed but mainly uncomplicated and one (*n* = 1089) consisted of women with diabetes.

Baseline and ultrasound characteristics of participants in the studies that contributed to the IPD are shown in Table 1. Mean GA at ultrasound was 37.2 weeks (SD 3.4), and mean EFW centile was 54.5 (SD 29.9).

**Table 1:** Baseline and ultrasound characteristics of participants in studies that contributed to the IPD

Baseline characteristics	Number of studies	Number of women	Mean (SD) or *n* (%)*
Age (years)	17	21 661	30.4 (6.1)
Body mass index (kg/m^2^)	14	19 239	25.5 (6.2)
**Ethnicity**			
Caucasian	12	18 274	10 842 (59.3%)
Black	12	18 274	2394 (13.1%)
Asian	12	18 274	3116 (17.1%)
Other	12	18 274	1992 (10.5%)
**Smoker**	13	19 870	2143 (10.8%)
**Nulliparous**	16	21 318	10 864 (51.0%)
**Pre‐eclampsia**	7	4085	376 (9.2%)
**Hypertension**	7	7419	729 (9.8%)
**Maternal diabetes**	9	7827	1916 (24.5%)
**Gestational age at ultrasound (weeks)**	17	21 647	37.2 (3.4)
**UA (pulsatility index)**	17	21 661	0.91 (0.34)
**MCA (pulsatility index)**	17	21 661	1.54 (0.41)
**Cerebroplacental ratio**	17	21 661	1.80 (0.56)
**EFW (g)**	8	6556	2639.0 (666.2)
**EFW centile**	8	6556	54.5 (29.9)

* Percentage of number of cases.

### Quality of included studies

No important issues were identified in checking IPD. Detailed results of the QUADAS‐2 risk of bias assessment are provided in the Supplementary material (Appendix S5). High or unclear risk of bias or suboptimal reporting was detected in 14/17 studies (82%). In nine studies (53%) it was unclear whether the obstetrician was blinded for the test results, and in five studies (29%) they were not blinded (details described in the Supplementary material, Appendix S4b). Subgroup analysis on the three blinded studies was not possible, because the main outcome was not provided. Only the accompanying study of one data set^25^ was described to have used CPR in diagnosing FGR. This data set was not used in the primary analysis, because the main outcome was not provided.

### Outcomes

Mean GA at delivery was 39.3 weeks (SD 2.5), with 1921 (8.9%) preterm deliveries. Mean BW centile was 47.1 (32.3), with 3594 (18.2%) cases <10th centile. All three components of the main, composite outcome were provided in 12/17 data sets (*n* = 18 731). Adverse outcome was observed in 3423 women (18.3%). When assessing the outcomes separately, perinatal death had occurred in 121 (0.6%) women (including 35 cases of stillbirth), emergency CS for fetal distress in 1696 women (7.9%) and neonatal unit admission in 2378 women (12.2%). More details of outcome measures can be found in the Supplementary material (Appendix S6).

**Figure 1:**
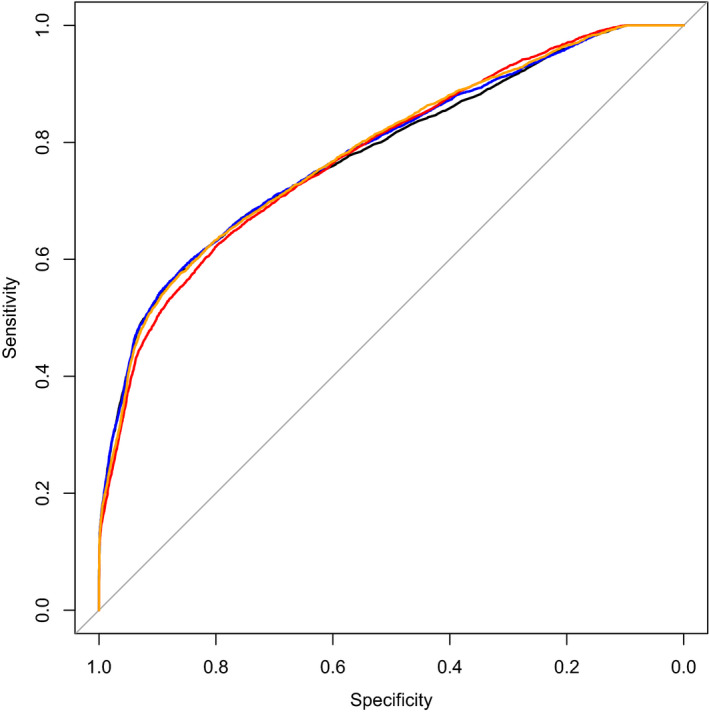
ROC curves for the prediction of adverse perinatal outcome of UA PI (black), MCA PI (red), CPR (orange) and UA PI plus CPR combined (blue). Corresponding AUCs: for UA PI 0.775 (95% CI 0.709–0.828), for MCA PI 0.773 (95% CI 0.709–0.826), for CPR 0.778 (95% CI 0.715–0.831) and for UA PI plus CPR combined 0.778 (95% CI 0.714–0.831).

All three tests (UA PI, MCA PI and CPR) were significantly associated with the main outcome, with odds ratios of 5.71 (95% CI 4.48–7.28) for UA PI, 0.55 (95% CI 0.48–0.63) for MCA PI and 0.49 (95% CI 0.44–0.55) for CPR. Addition of CPR to the UA PI model at logistic regression analysis resulted in a statistically significant increased goodness‐of‐fit (*P* < 0.001). However, the corresponding increase in discriminative ability was minimal, with a ΔAUC of 0.003. This is further visualised in Figure 1, which shows highly similar ROC curves, also of MCA PI and CPR. Separate analyses for the individual included studies also showed similar results (see Supplementary material, Appendix S7).

**Figure 2:**
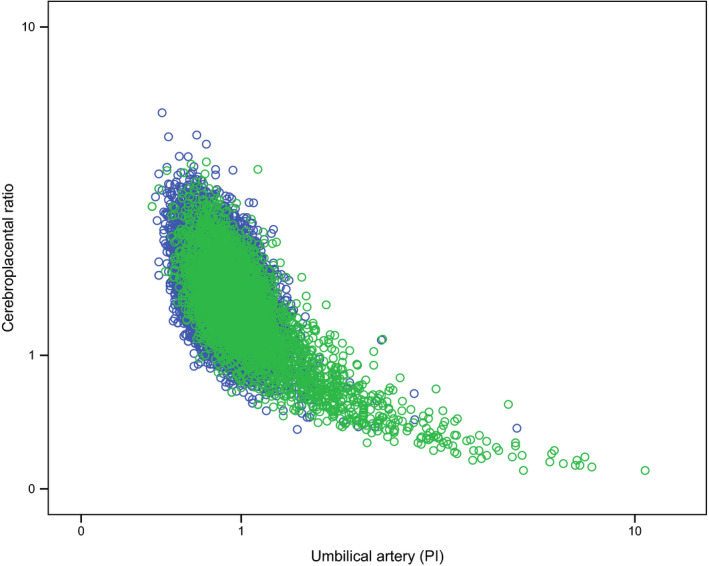
Scatter plot of cases with adverse outcome (green dots; *n* = 3423) and cases without adverse outcome (blue dots; *n* = 15 308) with their respective UA PI values (*x*‐axis, logarithmic) and CPR values (*y*‐axis, logarithmic).

**Figure 3:**
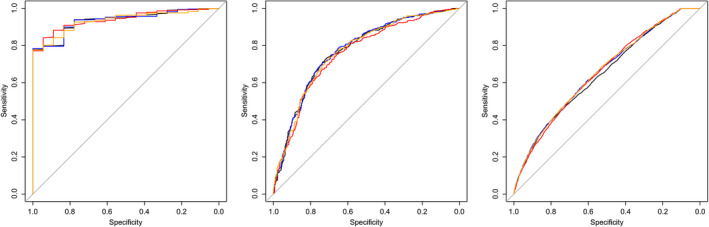
ROC curves in subgroups according to GA at delivery for the prediction of adverse perinatal outcome of UA PI (black), MCA PI (red), CPR (orange) and UA PI plus CPR combined (blue) for: GA at delivery <34 weeks (*n* = 836), GA at delivery 34–37 weeks (*n* = 1085) and GA at delivery ≥37 weeks (*n* = 19 704).

Figure 2 shows a scatterplot of the measurement results of participants with an adverse main outcome and those without. Here, it can be observed that participants with an adverse outcome sometimes had low CPR and high UA PI values. It also shows that there are no outliers to the bottom left of the figure of participants with an adverse outcome. In Appendix S8, Figures 1–3 illustrate the relation between MCA and CPR and between UA and MCA.

### Subgroup analyses

#### Gestational age at delivery

In all three subgroups, the increase in discriminative ability was minimal when adding CPR to UA PI alone, as visualised by the highly similar ROC curves (Figure 3) with ΔAUCs in subgroup 1 of 0.000 (GA < 34 weeks), in subgroup 2 of 0.002 (GA 34–37 weeks) and in subgroup 3 of 0.009 (GA ≥ 37 weeks) (Appendix S9: Table 1). ROC curves of all three tests separately (UA PI, MCA PI and CPR) were highly comparable, with ΔAUCs ranging from 0.000 to 0.020. Figure 3 also shows that the ROC curves of all three tests decreased with increasing GA.

#### Birthweight centile

**Figure 4:**
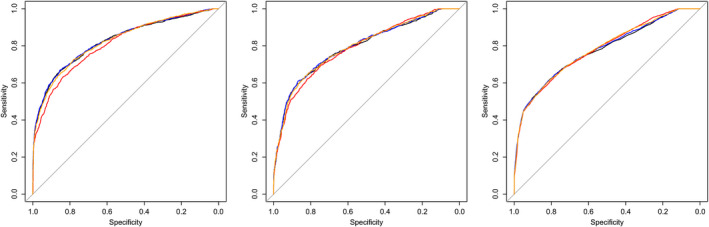
ROC curves in subgroups according to BW centile for the prediction of adverse perinatal outcome of UA PI (black), MCA PI (red), CPR (orange) and UA PI plus CPR combined (blue) for: BW centile <10 (*n* = 3594), BW centile between 10 and 25 (*n* = 2929) and BW centile ≥25 (*n* = 13 193).

In all three subgroups, the increase in discriminative ability was minimal when adding CPR to UA PI alone, as visualised by the highly similar ROC curves (Figure 4) with ΔAUCs in subgroup 1 of 0.004 (BW centile <10), in subgroup 2 of 0.004 (BW centile ≥10) and in subgroup 3 of 0.005 (BW centile ≥25) (see Supplementary material, Appendix S9: Table 2). ROC curves of all three tests separately (UA PI, MCA PI and CPR) were highly comparable, with ΔAUCs ranging from 0.000 to 0.022. Figure 4 also shows that ROC curves were highest for cases with BW centile <10 and slightly lower for cases with BW centile ≥10 and ≥25.

#### Estimated fetal weight centile

**Figure 5:**
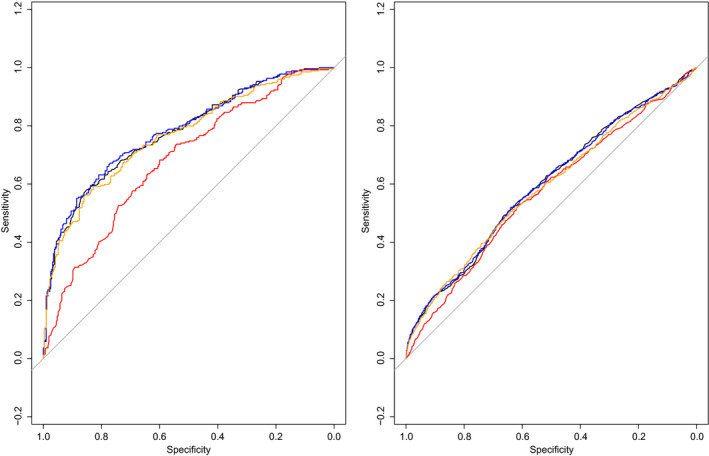
ROC curves in subgroups according to EFW centile for the prediction of adverse perinatal outcome of UA (black), MCA (red), CPR (orange) and UA PI plus CPR combined (blue) for: EFW centile <10 (*n* = 589) and EFW centile ≥10 (*n* = 3995).

In both subgroups the increase in discriminative ability was minimal when adding CPR to UA PI alone, as visualised by the highly similar ROC curves (Figure 5) with ΔAUCs in subgroup 1 of 0.004 (EFW centile <10) and in subgroup 2 of 0.000 (EFW centile ≥10). In cases with EFW centile <10, the ROC curve of MCA PI was lower than that of both UA PI and CPR (ΔAUC 0.096 and 0.085, respectively). In cases with EFW centile ≥10, ROC curves of all three tests separately (UA PI, MCA PI and CPR) were highly comparable, with ΔAUCs ranging from 0.009 to 0.025. Figure 5 also shows that ROC curves were highest for cases with EFW centile <10 and slightly lower for cases with EFW centile ≥10.

### Sensitivity analyses

Results were consistent across all sensitivity analyses, which can be found in the Supplementary material (Appendix S10: Tables 1–9, Figures 1–3). In no case did we observe a large increase of discriminative ability when CPR was added to UA PI alone.

### Unavailable studies

We were not able to obtain data from the authors of 34 (62%) out of 55 eligible studies. A list with the references of these studies and reasons for unavailability is provided in the Supplementary material (Appendix S11). Authors of half of these studies did not respond to our request to share their data for the IPD, despite perseverance. Authors of the other half of the studies were not able or willing to share their data, for which they provided different reasons, most often applying to logistical problems. We explored comparing the results of the unavailable studies with the main IPD results. Several factors, however, precluded a formal comparison. First, none of the studies used the same approach as the IPD, i.e. none of the studies presented results of the added value of CPR to UA PI alone. Second, in 15 studies only the MCA was investigated, and data of CPR could therefore not be extracted. Third, data were not extractable from another five of the 19 studies on CPR. Conclusions of the unavailable studies on CPR (*n* = 19), were positive in ten (53%), negative in six (32%) and unclear in three (16%) (see Supplementary material, Appendix S11a). As a comparison, in the included studies, conclusions were positive in nine (53%), negative in two (12%) and unclear in six (35%) (see Supplementary material, Appendix S4 and S3b). Also, we compared the reported accuracy estimates between the unavailable studies and the included studies (see Supplementary material, Appendix S11c), which showed no striking differences.

## Discussion

### Main findings

In this meta‐analysis of IPD involving singleton pregnancies, there was limited added value of CPR, when added to the well‐established UA Doppler measurement for prediction of adverse perinatal outcome. Our results consistently showed no difference in ROC curves, which means that whatever cutoff would be selected for a combination of CPR to the UA PI, similar discrimination can be obtained when relying on UA PI only. These results were consistent across all subgroup analyses, defined by GA and fetal size, and across all sensitivity analyses. The three Doppler measurements (UA PI, MCA PI and CPR) showed highly comparable predictive value. In early preterm pregnancies (delivery before 34 weeks of gestation), we observed the highest predictive value; in term pregnancies, predictive value was low. In small fetuses, predictive value was slightly higher than in normally grown fetuses.

#### Strengths and limitations

The main strength is that we combined IPD from multiple studies, based on a protocol developed in advance, which enabled us to perform data checks and multivariate analyses in predefined subgroups. This careful collaborative reanalysis of raw continuous data is the first to directly investigate the added predictive value of CPR to the established UA PI. Most previous studies assessed Doppler measurements as dichotomous variables, but cutoff points of CPR vary largely in both literature and practice.^26^ In our analyses, we therefore used the actually recorded continuous PI values without grouping. Another strength is the large heterogeneity of included participants, which enabled us to perform subgroup analyses. The large total sample size provided adequate power to detect small differences between AUCs in the overall study population for the main outcome.

A number of limitations deserve consideration. First, we were not able to include over half of the eligible studies, despite perseverance. Unwillingness of primary study authors to share IPD is a known problem.^27^, ^28^, ^29^, ^30^ Underlying reasons for unwillingness could be that authors are more keen to supply data from studies with promising results and reluctant to supply data from studies that were less encouraging – or vice versa. Also, authors may have been more hesitant to share data, because of the relative novelty of IPD and unfamiliarity of investigators with this type of research. In this IPD meta‐analysis, it was not possible to formally compare the study results with the results of the unavailable studies. Nonetheless, no striking differences were found when comparing the reported accuracy estimates between the unavailable studies and the included studies.

Another limitation is that half of the included data sets had been collected retrospectively. Also, many of the included studies were scored as being at risk of bias, which was primarily due to the fact that in only three out of 17 included data sets were obstetricians explicitly blinded for all Doppler results during clinical management. Nonetheless, most of the non‐blinded studies explained that CPR results were not used in clinical management, often because CPR results had been retrospectively calculated and had therefore been unknown during pregnancy and delivery. Still, inadequate blinding may have caused inappropriate obstetric interventions, and these interventions (e.g. early delivery) can in turn affect the rates of the adverse outcomes (e.g. NICU admission, CS for fetal distress) they are meant to predict or prevent.

The effect of intrapartum management on the outcome variables is another potential bias for all three investigated Doppler tests. It has been shown before that the often used outcomes CS for fetal distress and NICU admission are more influenced by intrapartum variables than either fetal size or Doppler values.^31^ Combining outcomes to create a composite outcome score could have potentially underestimated the effect of the investigated Doppler tests for outcomes that are not influenced by labour, such as stillbirth. To investigate this effect, we performed sensitivity analyses for stillbirth and in women with only elective CS. This consistently led to the same results. In general, the large variation in outcome reporting across studies of FGR remains a complicating factor in comparing different study results.^32^ For this reason, a core outcome set for growth restriction is currently being developed.^33^

#### Interpretation

Our results are in line with a recently published large prospective observational study by Akolekar et al.^34^ The authors had measured the CPR in 47 211 singleton pregnancies undergoing routine ultrasound examination at 35–37 weeks of gestation and investigated the predictive value of CPR for a composite adverse perinatal outcome. The authors found low likelihood ratios in normally grown and growth‐restricted fetuses, and concluded that the performance of CPR in the prediction of each adverse outcome was poor, independent of fetal size or interval between testing and delivery.

Comparing the results of this study with previous studies is impeded by its different design, comparing the added value of CPR with the existing UA PI in continuous data, instead of assessing CPR as a stand‐alone, dichotomised test. Our results confirm findings from multiple previous studies that CPR and MCA PI have discriminative ability, whereas this was not found to be stronger than that of UA PI. In the term period, CPR and MCA PI did not become abnormal more often than UA PI throughout the continuous data in our study, in contrast to previous observations. A possible explanation could be variation between the tests’ cutoff values.^25^ The findings of this IPD provide an answer to the inconclusive findings of our previous systematic review,^8^ as most of its limitations – such as suboptimal reporting of inclusion criteria, large heterogeneity in outcome reporting and the use of different test cutoff values across studies – were overcome in this IPD.

More research is needed to optimise fetal diagnosis and monitoring, specifically regarding clinical management strategies, including cutoff values for Doppler measurements. The next step could be the development of an individualised prediction model for adverse perinatal outcome, taking into account all relevant factors (e.g. GA, fetal size and growth, Doppler measurements and maternal factors). Ultimately, such a model could be used to aid in deciding the timing of delivery for each individual woman.

## Conclusion

In this IPD meta‐analysis with continuous data, Doppler measurement of CPR added no predictive value for adverse perinatal outcome beyond UA PI, when assessing the fetal condition in singleton pregnancies, irrespective of GA or fetal size. Predictive value of Doppler measurements of the UA PI, MCA PI and CPR was comparable and highest in preterm fetuses, but there appeared to be a more limited role for Doppler ultrasound in monitoring term pregnancies with normal fetal size. The findings in this IPD meta‐analysis do not support the use of CPR outside a research setting. We believe future research should focus on improving clinical management strategies combining all relevant factors, and on the development of individualised prediction models for pregnancies at high‐risk of placental insufficiency.

### Disclosure of interests

BWM reports grants from NHMRC and personal fees from ObsEva, Merck Merck KGaA, Guerbet and iGenomix outside the submitted work. The other authors report no disclosures of interest. Completed disclosure of interest forms are available to view online as supporting information.

### Contribution to authorship

CVHS, BWM, PB and MB designed the study in consultation with CG and CB. CVHS and LS performed the literature search. Subsequently, research groups of eligible reports were contacted and invited by CVHS and IO to participate in the IPD meta‐analysis and to send the raw data of their study. All contributing authors became part of the CPR IPD Study Group. Data extraction, and range and consistency checks, were performed on the received data sets by CVHS and IO. MH and CVHS conducted the data analyses, in consultation with PB. CVHS wrote the initial and subsequent drafts of the manuscript; WG and all other authors made critical revisions to the manuscript.

### Details of ethics approval

Not applicable.

### Funding

None.

### Acknowledgement

We would like thank Dr F. Figueras, Prof. E. Gratacós, Dr F. Crispi and Dr J. Miranda for sharing data for this project.

**The CPR IPD Study Group:** Asma Khalil (Fetal Medicine Unit, St George’s Hospital Medical School and St George’s University of London, London, UK; Vascular Biology Research Centre, Molecular and Clinical Sciences Research Institute, St George’s University of London, London, UK), Basky Thilaganathan (Fetal Medicine Unit, St George’s Hospital Medical School and St George’s University of London, London, UK; Vascular Biology Research Centre, Molecular and Clinical Sciences Research Institute, St George’s University of London, London, UK), Ozhan M Turan (Departments of Obstetrics, Gynecology and Reproductive Sciences, University of Maryland School of Medicine, Baltimore, MD, USA), Sarah Crimmins (Departments of Obstetrics, Gynecology and Reproductive Sciences, University of Maryland School of Medicine, Baltimore, MD, USA), Chris Harman (Departments of Obstetrics, Gynecology and Reproductive Sciences, University of Maryland School of Medicine, Baltimore, MD, USA), Alisson M Shannon (Departments of Obstetrics, Gynecology and Reproductive Sciences, University of Maryland School of Medicine, Baltimore, MD, USA), Sailesh Kumar (School of Medicine, The University of Queensland, Brisbane, QLD, Australia; Mater Research Institute – University of Queensland, South Brisbane, QLD, Australia), Patrick Dicker (Department of Epidemiology and Public Health, Royal College of Surgeons in Ireland), Fergal Malone (Departments of Obstetrics and Gynaecology, Royal College of Surgeons in Ireland), Elizabeth C Tully (Departments of Obstetrics and Gynaecology, Royal College of Surgeons in Ireland), Julia Unterscheider (Department of Maternal Fetal Medicine, The Royal Women’s Hospital, Melbourne, VIC, Australia), Isabella Crippa (Department of Obstetrics and Gynaecology, University of Milano‐Bicocca, Monza, Italy), Alessandro Ghidini (Department of Obstetrics and Gynaecology, University of Milano‐Bicocca, Monza, Italy), Nadia Roncaglia (Department of Obstetrics and Gynaecology, University of Milano‐Bicocca, Monza, Italy), Patrizia Vergani (Department of Obstetrics and Gynaecology, University of Milano‐Bicocca, Monza, Italy), Amarnath Bhide (Fetal Medicine Unit, St George’s Hospital Medical School and St George’s University of London, London, UK), Francesco D'Antonio (Fetal Medicine Unit, St George’s Hospital Medical School and St George’s University of London, London, UK), Gianluigi Pilu (Policlinico S. Orsola‐Malpighi, University of Bologna, Bologna, Italy), Alberto Galindo (Fetal Medicine Unit‐SAMID, Department of Obstetrics and Gynaecology, University Hospital 12 de Octubre, 12 de Octubre Research Institute (imas12), Complutense University of Madrid, Madrid, Spain), Ignacio Herraiz (Fetal Medicine Unit‐SAMID, Department of Obstetrics and Gynaecology, University Hospital 12 de Octubre, 12 de Octubre Research Institute (imas12), Complutense University of Madrid, Madrid, Spain), Alicia Vázquez‐Sarandeses (Fetal Medicine Unit‐SAMID, Department of Obstetrics and Gynaecology, University Hospital 12 de Octubre, 12 de Octubre Research Institute (imas12), Complutense University of Madrid, Madrid, Spain), Cathrine Ebbing (Department of Obstetrics and Gynaecology, Haukeland University Hospital, Bergen, Norway), Synnøve L Johnsen (Department of Obstetrics and Gynaecology, Haukeland University Hospital, Bergen, Norway), Henriette O Karlsen (Research Group for Pregnancy, Fetal Development and Birth, Department of Clinical Science, University of Bergen, Bergen, Norway).

## Supporting information


**Appendix S1.** Search strategy.
**Appendix S2.** Study selection.
**Appendix S3.** Data changes.
**Appendix S4.** (a) Characteristics of data sets included in the IPD. (b) Blinding of study results and conclusion scoring of data sets included in the IPD. (c) References of accompanying studies to data sets included in the IPD.
**Appendix S5.** Results of QUADAS‐2 assessment of risk of bias and applicability concerns.
**Appendix S6.** Details of outcome measures reported in studies that contributed to the IPD.
**Appendix S7.** Areas‐under‐the‐curves, in each included individual study, of UA PI, MCA PI, CPR and CPR added to UA PI for the composite adverse perinatal outcome. Data sets 4, 10 and 15–17 were not included here, as the composite adverse outcome was not provided.
**Appendix S8.** Scatter plots.
**Appendix S9.** Subgroup analyses presented in tables.
**Appendix S10.** Sensitivity analyses.
**Appendix S11.** (a) Studies not included in the IPD. (b) References of studies not included in the IPD. (c) Reported accuracy estimates of CPR in studies unavailable for the IPD versus in studies included in the IPD.
