# Supplementary material for: Cerebroplacental ratio in predicting adverse perinatal outcome: a meta‐analysis of individual participant data
Source: BJOG. 2020 Jun 8;128(2):226–35. doi: 10.1111/1471-0528.16287 (PMC7818434; doi:10.1111/1471-0528.16287)

Appendix S1. Search strategy.

Performed on June 26 2015, updated on July 11 2017.

1. PubMed – 2070 citations
    1. "Pregnant Women"[Mesh] OR "Pregnancy"[Mesh] OR "Fetus"[Mesh] OR pregnan*[tiab] OR fetus*[tiab] OR fetal[tiab] OR foetal[tiab] OR foetus*[tiab] OR perinatal[tiab]
    2. ("Middle Cerebral Artery"[Mesh] OR MCA[tiab] OR (cerebral[tiab] AND (artery[tiab] OR arteries[tiab] OR blood flow[tiab]))) AND ("Ultrasonography, Doppler"[MeSH] OR "Ultrasonography, Prenatal"[MeSH] OR "Prenatal Diagnosis"[MeSH] OR "blood flow velocity"[MeSH] OR doppler[tiab] OR ultrasonograph*[tiab] OR ultrasound[tiab] OR sonograph*[tiab] OR imaging[tiab] OR echograph*[tiab]) OR cerebroplacental[tiab] OR cerebro placental[tiab] OR cerebroumbilical[tiab] OR cerebro umbilical[tiab] OR brain sparing[tiab] OR brainsparing[tiab] OR (cerebral[tiab] AND redistribution[tiab]) OR "blood flow redistribution"[tiab]
    3. #1 AND #2
    4. #3 NOT ("Animals"[Mesh] NOT "Humans"[Mesh])
2. EMBASE.com – 2871 citations
    1. 'pregnant woman'/exp OR 'pregnancy'/exp OR 'fetus'/exp OR pregnan*:ab,ti OR fetus*:ab,ti OR fetal:ab,ti OR foetal:ab,ti OR foetus*:ab,ti OR perinatal:ab,ti
    2. ('middle cerebral artery'/exp OR MCA:ab,ti OR (cerebral:ab,ti AND (artery:ab,ti OR arteries:ab,ti OR 'blood flow':ab,ti))) AND ('Doppler echography'/exp OR 'fetus echography'/exp OR 'prenatal diagnosis'/exp OR 'blood flow velocity'/exp OR doppler:ab,ti OR ultrasonograph*:ab,ti OR ultrasound:ab,ti OR sonograph*:ab,ti OR imaging:ab,ti OR echograph*:ab,ti) OR cerebroplacental:ab,ti OR 'cerebro placental':ab,ti OR cerebroumbilical:ab,ti OR 'cerebro umbilical':ab,ti OR 'brain sparing':ab,ti OR brainsparing:ab,ti OR (cerebral:ab,ti AND redistribution:ab,ti) OR 'blood flow redistribution':ab,ti
    3. #1 AND #2
    4. #3 NOT ([animals]/lim NOT [humans]/lim)
3. Cochrane Library – 126 citations
    1. pregnan* OR fetus* OR fetal OR foetal OR foetus* OR perinatal:ti,ab,kw
    2. (MCA OR (cerebral AND (artery OR arteries OR "blood flow"))) AND (ultrasonograph* OR echograph* OR "prenatal diagnos*" OR "blood flow velocit*" OR doppler OR ultrasound OR sonograph* OR imaging) OR cerebroplacental OR "cerebro placental" OR cerebroumbilical OR "cerebro umbilical" OR "brain sparing" OR brainsparing OR (cerebral AND redistribution) OR "blood flow redistribution":ti,ab,kw
    3. #1 AND #2
4. ClinicalTrials.gov – 21 citations
    1. (pregnan* OR fetus OR foetus OR foetal OR fetal) AND ("middle cerebral artery" or MCA OR cerebroplacental OR "cerebro placental" OR cerebroumbilical OR "cerebro umbilical" OR "brain sparing" OR brainsparing OR (cerebral AND redistribution))

Appendix S2. Study selection.

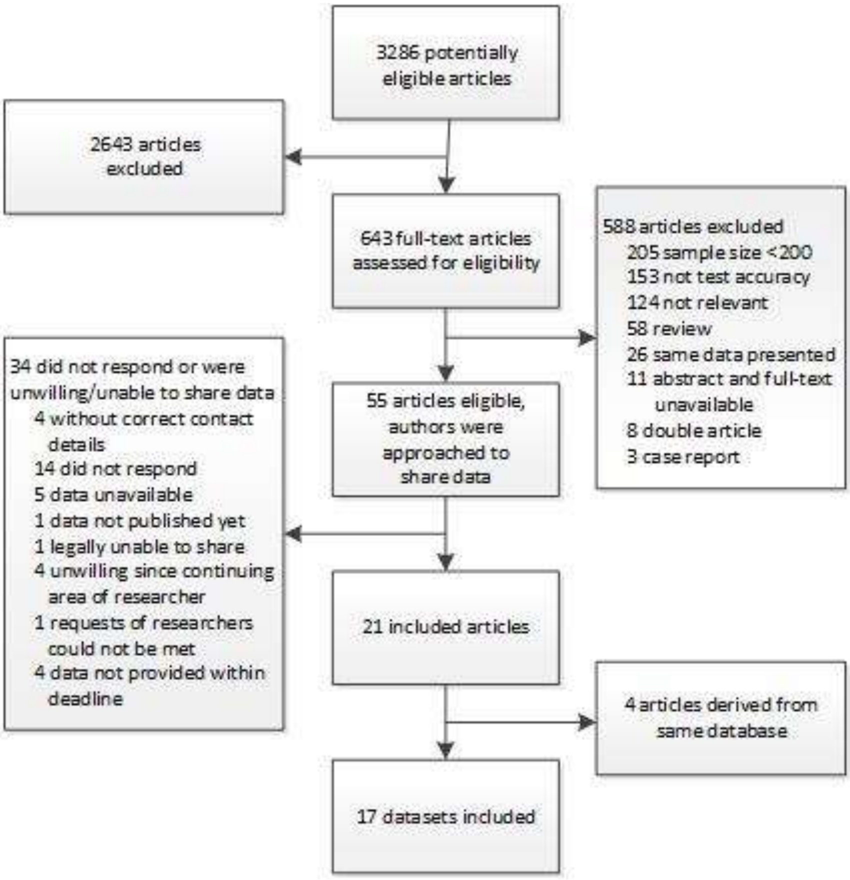

Appendix S3. Data changes.

Dataset	Change	Reason for change	ID
1	Deleted gestational age at delivery and time-to delivery	Obvious error (time-to-delivery negative value)	1140, 3593, 3830, 5671, 7431, 9199
2	Changed Maternal height 17500 into 175.00	Obvious error (decimals wrong)	183
	Changed Maternal height 17020 into 170.20	Obvious error (decimals wrong)	237
	Changed Maternal height 16000 into 160.00	Obvious error (decimals wrong)	316
	Changed MCA pulsatility index 106.00 into 1.06	Obvious error (decimals wrong)	33
	Deleted gestational age at delivery and time-to delivery	Obvious error (time-to-delivery negative value)	252, 344, 347
3	Corrected gestational age at delivery and time-to delivery according to due date	Obvious error (time-to-delivery negative value)	109, 210
	Deleted gestational age at delivery and time-to delivery	Obvious error (time-to-delivery negative value)	125
	Deleted BPD and HC values	Impossible and obvious error (same as AC and FL)	184
	Deleted BPD, HC, AC and FL values	Impossible values	120
4	Deleted gestational age at delivery	Obvious error (time-to-delivery negative value)	70, 125, 215, 240
5	Deleted gestational age at delivery and time-to delivery	Obvious error (time-to-delivery negative value)	461, 535, 507, 534, 532, 518, 237, 459
	Changed MCA pulsatility index 142.00 into 1.42	Obvious error (decimals wrong)	504
	Deleted 109 values of 999.99	Different coding for missing values	n=109
6	Deleted gestational age at delivery and time-to delivery	Obvious error (time-to-delivery negative value)	71
		No variables available	
	Excluded cases		35, 55, 57, 85, 132, 142, 508, 529, 533, 540,
		Dopplers not available	542, 547, 554
	Excluded cases		520, 544
7	Excluded cases	Dopplers not available	781, 795
	Excluded cases	Congenital defect	n=175
	Excluded case	"Anti-Kel"	375
	Excluded case	"Rh sensitized, fetal hemolytic anemia"	1193
	Excluded case	"RH Jaundice"	1059
	Excluded case	"HEMOLYTIC DZ"	793
	Deleted Maternal height and BMI values	Obvious error (impossible values)	596
	Deleted Maternal weight and BMI values	Obvious error (impossible values)	109, 635, 927
	Deleted MVP value (156)	Impossible value	115
	Deleted MVP value (802)	Impossible value	194
8	Changed Birthweight value of 12.6 into 1260	Obvious error (decimals wrong)	59
9	Excluded cases	Dopplers not available	n=73
	Excluded cases	Single umbilical artery	n=42

	Excluded case	Syndrome	914
	Deleted case	Impossible umbilical artery PI value	1262
	Deleted pH arterial value (1.1)	Impossible value	683
	Deleted pH arterial value (2.17) and pH venous value (2.2)	Impossible value	818
	Deleted pH arterial value (2.23) and pH venous value (2.29)	Impossible value	546
	Deleted pH arterial value (2.26)	Impossible value	744
	Deleted pH arterial value (8.26)	Impossible value	39
10	Changed variable maternal diabetes from “no” into “yes”	Stated at variable “Maternal condition” as “GDM”	387, 400
11	Deleted gestational age at delivery	Obvious error (time-to-delivery negative value)	816, 968, 1211
12	No changes		
13	No changes		
14	No changes		
15	Changed year 2011 into 2010	Obvious error (negative value)	269, 270
	Deleted maternal age value of 0.13 years	Obvious error (impossible value)	128
	Deleted Maternal height value	Obvious error (impossible value)	271
	Deleted gestational age at ultrasound of 52,14 and time-to delivery	Obvious error (impossible value and time-to-delivery negative value)	195
16	Excluded cases	Dopplers not available	n=72
	Excluded case	Chromosomal abnormality	551
	Deleted gestational age at delivery and time-to delivery	Obvious error (time-to-delivery negative value)	100, 167, 720
	Deleted FL value of 316.0	Impossible value	136
	Deleted HC values of 87.0 and 86.0	Impossible value	136, 338
	Deleted maternal age of -7014.022 years	Impossible value	727
	Deleted maternal age of 0.219 years	Impossible value	277
	Changed AC from 3016.0 into 301.6	Obvious error (decimals wrong)	337
	Changed maternal height of 165.0 into 1.65	Obvious error (decimals wrong)	890
	Changed drugs "1 aas" into "1"	Obvious error	497
	Changed Apgar score of "70" into "10"	Obvious error	524
	Deleted BMI value of 0.2	Impossible value	890
17	Deleted Apgar values of -99	Impossible value	n=90
	Changed year of delivery from 2006 into 2007	Obvious error (time-to-delivery negative value)	1050
	Deleted value “0” of Left Uterine artery	Impossible value	581
	Changed value “62” into “0.62” of Left Uterine artery	Impossible value, obvious error	232
	Changed AC values from “29.0” into 290”	Obvious error (decimals wrong)	297, 455
	Deleted AC value of “61”	Impossible value	488

	Deleted values AC and HC	Impossible value	760
	Deleted HC value	Impossible value	722
	Deleted HC and FL values	Impossible value	481
	Excluded cases	Dopplers not available	n=27 cases
	Changed AFI value from “75” into “7.5”	Obvious error (decimals wrong)	494

Appendix S4a. Characteristics of datasets included in the IPD.

Dataset #	Accompanying report	Country	Design	Setting	Outcomes provided in dataset	Cases sent	Cases used	Reason(s) for exclusion of cases	All three components of the composite adverse perinatal outcome provided
1	Khalil 2015[1], Khalil 2016[2]	UK	Retrospective cohort	Morphologically normal fetuses born at term that had an ultrasound scan <2 weeks of delivery. (2016 et al.: The indications for the ultrasound assessment performed in the third trimester included suspected poor/excessive fetal growth, reduced fetal movements, history of SGA or large-for-gestational-age baby, high mid-trimester UtA Doppler indices or gestational diabetes. Therefore, by definition, these pregnancies were at risk of fetal growth disorders.) Exclusion criteria: fetal abnormality, aneuploidy or genetic syndrome, or stillbirth.	Perinatal death, emergency cesarean for fetal distress, NNU admission	9215	9215	-	Yes
2	D’Antonio 2013[3]	UK	Retrospective cohort	Uncomplicated pregnancies reaching 41 weeks’ gestation	Perinatal death, emergency cesarean for fetal distress, NICU admission, Apgar score, arterial cord blood pH	406	321	Dopplers not provided (n=85)	Yes
3	Vázquez-Sarandeses 2016[4]	Spain	Retrospective cohort	Singleton pregnancies with EFW<10th centile identified ≥ 32+0 weeks gestation	Perinatal death, emergency cesarean for fetal distress, NICU admission, Apgar score, arterial cord blood pH	223	198	Multiple pregnancies (n=24), Dopplers not provided (n=1)	Yes

4	Severi 2002[5]	Italy	Retrospective cohort	Singleton pregnancy with a birth weight < 10th centile; absence of severe maternal complications; normal umbilical artery Doppler; and availability of complete follow-up	Perinatal death, emergency cesarean for fetal distress	241	241	-	No
5	Vergani 2010[6]	Italy	Prospective cohort	FGR (AC <10th centile) cases delivered ≥34 weeks, ultrasound within 2 weeks of delivery	Perinatal death, emergency cesarean for fetal distress, NICU admission, Apgar score, arterial cord blood pH	481	459	Dopplers not provided (n=2)	Yes
6	Karlsen 2016[7]	Norway	Prospective cohort	Referred for a 24-week US due to increased risk of having an SGA, and patients diagnosed with an SGA fetus (≤5th centile)	Perinatal death, emergency cesarean for fetal distress, Apgar score, NICU admission	207	205	Dopplers not provided (n=2)	Yes
7	Crimmins 2016[8], Shannon 2017[9]	USA	Retrospective cohort	Singleton non-anomalous pregnancies delivered >34 weeks with EFW appropriate for GA and middle cerebral artery (MCA) Doppler assessed within 14 days of birth.	Perinatal death, emergency cesarean for fetal distress, NICU admission, Apgar score, arterial cord blood pH	1196	1015	Congenital abnormalities (n=175)<br>Dopplers not provided (n=2)<br>Fetal hemolytic anemia (n=4)	Yes
8	Manogura 2008[10]	USA	Prospective cohort	Placental-based IUGR (abdominal circumference <p5, abnormal UA Doppler), delivery <37 weeks, exclusion if intra-uterine fetal death	Perinatal death, emergency cesarean for fetal distress, NICU admission, Apgar score, arterial cord blood pH	404	404	-	Yes
9	Jain 2011[11]	USA	Retrospective cohort	Third trimester antepartum surveillance	Perinatal death, emergency cesarean for fetal distress, NICU admission, Apgar score, arterial cord blood pH	2754	2072	Dopplers not provided (n=73)<br>Congenital abnormalities (n=43)<br>No follow-up (n=565)<br>Impossible Doppler value (n=1)	Yes
10	Bligh 2018[12], Bligh 2016[13]	Australia	Prospective cohort	Uncomplicated singleton pregnancies underwent fortnightly CPR measurement from 36 weeks to delivery	Emergency cesarean for fetal distress, NICU admission, low Apgar score, acidosis (NB: the authors defined acidosis as pH<7.1 and/or lactate≥6mmol/L)	437	437	-	No
11	Flatley 2017[14]	Australia	Retrospective cohort	Women with a non-anomalous singleton fetus with at least two ultrasound scans between 30+0 and 37+0 weeks. Exclusion criteria were major congenital abnormality,<br>aneuploidy, multiple pregnancy and unknown MCA PI or UA PI.	Perinatal death, emergency cesarean for fetal distress, acidosis, NCCU admission (included admission to the special care nursery (SCN), intensive care nursery	1693	1693	-	Yes

					(ICN) and intensive care unit (ICU))				
12	Gibbons 2017[15]	Australia	Retrospective cohort	Women with diabetes mellitus (pre-existing insulin dependent diabetes (pT1DM) mellitus, pre-existing non-insulin dependent diabetes mellitus (pT2DM) and gestational diabetes mellitus (GDM) with measurement of the CPR between 34+0 and 36+6 weeks.	Perinatal death, emergency cesarean for fetal distress, Apgar score, cord blood pH (NB: the authors defined acidosis as pH ≤ 7.0 or lactate > 6 mmol/L), NCCU admission (included admission to the special care nursery (SCN), intensive care nursery (ICN) and intensive care unit (ICU))	1089	1089	-	Yes
13	Twomey 2016[16]	Australia	Retrospective cohort	Women with non-anomalous, singleton pregnancies. Exclusion criteria included multiple pregnancy, known genetic conditions or congenital malformations, non-cephalic presentation, ruptured membranes, absent/reversed end-diastolic flow in the UA, unknown UA PI or MCA PI, or unknown mode of delivery. Indications for requesting a fetal growth and wellbeing scan at 30–34 weeks varied, although the commonest reasons were uncertainty of fetal size or presentation on clinical examination, previous pregnancy complications or maternal anxiety.	Perinatal death, emergency cesarean for fetal distress, NCCU admission (included admission to the special care nursery (SCN), intensive care nursery (ICN) and intensive care unit (ICU))	1224	1224	-	Yes
14	Unterscheider 2013[17], Flood 2014[18]	Ireland	Prospective cohort	PORTO study: IUGR, EFW >500 g, GA at test 24-37 weeks	Perinatal death, emergency cesarean for fetal distress, NICU admission, Apgar score, arterial cord blood pH	881	881	-	Yes
15	Mula 2013[19]	Spain	Prospective cohort	Singleton pregnancies at routine third-trimester scan (32–35.6 weeks). Inclusion criteria: (i) EFW >10 centile and (ii) UA PI <p95. Exclusion criteria: (i) congenital malformations, (ii) BW <p10, or (iii) maternal diseases, including diabetes, chronic hypertension, autoimmune and other systemic diseases.	Apgar score, acidosis, NICU admission	258	258	-	No
16	Triunfo 2017[20]	Spain	Prospective cohort	Low-risk singleton pregnancies scanned at 37 gestational weeks	Emergency cesarean for fetal distress, Apgar score, arterial cord blood pH	998	926	Dopplers not provided (n=72)<br>Chromosomal abnormality (n=1)	No

17	Miranda 2017[21]	Spain	Nested case-control study within a prospective cohort	Singleton gestations attending their routine third-trimester evaluation (32+0 – 36+6 weeks of gestation), including women who delivered a neonate with BW <10th centile (n=175) and a group of controls, in a ratio of 5:1 (n=875), comprising consecutive uncomplicated pregnancies in the same period, matched for GA at scan (±2 weeks).	Perinatal death, emergency cesarean for fetal distress, Apgar score, arterial cord blood pH	1050	1023	Dopplers not provided (n=27)	No

Appendix S4b. Blinding of study results and conclusion scoring of datasets included in the IPD.

**Dataset #**	**Accompanying report**	**Was the clinician blinded for the Doppler results?**	Citation	**Was clinical manegement based on CPR?**	Citation	**Degree of conclusion positivity**	Citation #1	Citation #2
1	Khalil 2015[1], Khalil 2016[2]	No	"The results of the ultrasound and Doppler assessment were not blinded, giving rise to the possibility of subsequent clinical intervention and a 'treatment effect.' However, during the study period, intervention in the form of induction of labor was only undertaken for EFWof <5th centile or UA PI >95th centile, as per local protocol. The neonatologists were not blinded to the size, while they were to CPR values."	No	"During the study period, intervention in the form of induction of labor was only undertaken for EFW of <5th centile or UA PI >95th centile, as per local protocol. Hence the relations among fetal Doppler findings, BW, and labor outcomes should be relatively uninfluenced by these interventions."	Positive	"Lower fetal CPR, regardless of the fetal size, was independently associated with the need for operative delivery for presumed fetal compromise and with NNU admission at term. The extent to which fetal hemodynamic status could be used to predict perinatal morbidity and optimize the mode of delivery merits further investigation."	"Third-trimester CPR is an independent predictor of stillbirth and perinatal mortality. The role of UtA Doppler, CPR and EFW in assessing risk of adverse pregnancy outcome should be evaluated prospectively."
2	D'Antonio 2013[3]	No	Of these, 18 women had Doppler information available; two women underwent immediate induction of labor owing to elevated UA-PI. Etc.	No	Immediate induction of labor was arranged if the estimated fetal weight was below the 5th centile for gestational age, if anhydramnios was present on ultrasound assessment or if computerized cardiotocography (CTG) criteria for normality were not met. Otherwise, induction of labor was scheduled for 42 completed weeks' gestation.	Negative	"CPR is not predictive of unfavorable outcome in women with pregnancies lasting more than 41 weeks."	

3	Vázquez-Sarandeses 2016[4]	Unclear	Retrospective study	Unclear	Not described	Not applicable / Neutral	In our population, the predictive risk model for adverse perinatal outcomes in late-onset SGA showed a predictive capability similar to that observed by the original study. According to this algorithm, the measurement of CPR, mUtA-PI and EFW in the evaluation of late-onset SGA is of moderate usefulness for predicting adverse perinatal outcome.	
4	Severi 2002[5]	Unclear	Retrospective study	Unclear	Not described	Positive	"SGA fetuses with normal umbilical artery Doppler waveforms and abnormal uterine arteries and fetal middle cerebral artery waveforms have an increased risk of developing distress and being delivered by emergency Cesarean section. Particularly when both uterine and fetal cerebral waveforms are altered at the same time, the risk is exceedingly high (86%) and delivery as soon as fetal maturity is achieved seems advisable. On the other hand, when both vessels have normal waveforms, the chances of fetal distress are small (4%) and expectant management is the most reasonable choice."	
5	Vergani 2010[6]	No	"UA PI >p95 was an indication for hospital admission and daily monitoring of fetal well-being, as well as induction of labor after 37.0 weeks. Results of Doppler waveform analysis of the MCA were not used for	No	"Results of Doppler waveform analysis of the MCA were not used for patient management. "	Not applicable / Neutral	"In late preterm or term FGR, GA at delivery is the most important predictor of adverse neonatal outcome. At >37.5 weeks, delivery may be the best option to minimize adverse outcome in all FGR cases. At 34–37 weeks, a score based on GA	

			patient management. Pregnancy outcome and neonatal follow-up were obtained in all cases from review of the patients' records. "				at delivery, UA-PI centile and AC centile optimally predicts adverse neonatal outcome."	
6	Karlsen 2016[7]	No	"Managing clinicians were not blinded for the Doppler findings. This may have biased the relationship with the adverse outcomes, whereas the conditional centiles for MCA PI and CPR were not available for clinicians, and therefore were a less likely source of bias."	No	"In line with local and national guidelines, the finding of "brain sparing," i.e. MCA PI or CPR ≤5th centile, prompted closer surveillance or delivery in late preterm and term pregnancies in our department, and managing clinicians were not blinded for the Doppler findings. "	Positiv e	"Conditional centile for middle cerebral artery pulsatility index and cerebroplacental ratio ≤5 and ≤10 are associated with adverse perinatal outcomes. When adding conditional centile to conventional centile for cerebroplacental ratio, the prediction improved compared with the use of conventional centile alone."	
7	Crimmins 2016[8], Shannon 2017[9]	Unclear	Retrospective study	Uncle ar	Not described	Positiv e	"In this study of fetuses with normal-range EFW, multivessel Doppler cerebral blood flow abnormalities predict NICU admission, low weight at delivery, and risk of stillbirth. These relationships are true regardless of the EFW category. This data appears to validate use of multi-vessel Doppler in third trimester assessment of normal-sized fetuses."	"When brain sparing is observed, the greatest risk for stillbirth occurs in non-diabetic women with a normal weight fetus. The plausibility of this finding is that current surveillance protocols for diabetics are much more aggressive than in the non-diabetic population. Therefore evidence of brain sparing may be used as a valuable tool for increased surveillance in an otherwise low risk population."
8	Manogura 2008[10]	Unclear	Retrospective study	Uncle ar	Not described	Not applica ble / Neutral	"Placental disease predisposes the severely growth-restricted neonate to necrotizing enterocolitis. Even when arterial and venous Doppler variables are taken into consideration, birthweight remains the predominant risk factor for NEC. Further research should focus on the critical transition to neonatal life to	

							identify relevant triggers in predisposed neonates."	
9	Jain 2011[11]	Unclear	Retrospective study	No	"In our center, standard monitoring includes UA, MCA, non-stress testing and five component biophysical profile score (BPS Manning)."	Not applicable / Neutral	"Cerebrouterine ratio (CUR) predicts adverse outcome more often than CPR alone. Clinical studies using these complimentary tests are in order to evaluate the clinical impact of prediction of near-term compromise."	
10	Bligh 2018[12], Bligh 2016[13]	Yes	"Women and clinicians were blinded to the ultrasound results. The only exceptions for disclosing ultrasound findings were malpresentation, severe oligohydramnios (deepest pool <1cm) or absent or reversed flow in the umbilical artery, as these findings would influence immediate obstetric management."	No	Blinded	Positive	"The CPR 10th centile may be useful as a component of a risk assessment tool for Cesarean section for IFC in low risk pregnancies at term."	"Fetuses that have lower CU ratios from 36weeks onwards are at greater risk of becoming compromised in labour, requiring emergency delivery and being delivered in poorer condition. This may be useful in individualised risk assessment for fetal distress prior to labour."
11	Flatley 2017[14]	Unclear	Retrospective study	Unclear	Not described	Positive	"Our results suggest that both the individual CPR z-score and the magnitude and direction of change of CPR z-score can identify women whose pregnancies are at risk of various adverse perinatal outcomes, however, the CPR z-score at 35-37 weeks gestation appears to be a better predictor of outcomes."	
12	Gibbons 2017[15]	Unclear	Retrospective study	No	"Whilst it is possible that estimated fetal weight may have been considered when deciding mode of delivery, the CPR itself was unlikely to influence such decisions as this parameter was not included in the ultrasound report."	Positive	"Regardless of the type of DM, a low CPR was associated with poorer neonatal outcomes."	
13	Twomey 2016[16]	Unclear	Retrospective study	Unclear	Not described	Positive	"This study suggests that a CUR 1, measured at 30–34	

							weeks, is associated with a greater risk of emergency cesarean delivery for fetal compromise and a number of other adverse perinatal outcomes. The association was strongest in low birth weight babies."	
14	Unterscheider 2013[17], Flood 2014[18]	No	"A report of all sonographic findings was recorded in the patient's case file and was readily available to the managing clinician."	No	"Because the CPR was calculated retrospectively, this result was not made available to the clinician, and therefore, CPR results did not influence management decisions."	Positiv e	"Irrespective of the CPR calculation used, brain sparing is significantly associated with an adverse perinatal outcome in IUGR. This adds further weight to integrating CPR evaluation into the clinical assessment of IUGR pregnancies. The impact of this finding on longterm neurodevelopmental outcomes in this patient cohort is underway."	"In contrast to previous reports, we have demonstrated multiple potential patterns of Doppler deterioration in this large prospective cohort of IUGR pregnancies, which calls into question the usefulness of multivessel Doppler assessment to inform frequency of surveillance and timing of delivery of IUGR fetuses. These data will be critically important for planning any future intervention trials."
15	Mula 2013[19]	Yes	Deliveries were attended by a staff obstetrician blinded to the Doppler results.	No	Blinded	Not applica ble / Neutral	"Normally grown fetuses with increased frontal brain perfusion have poorer neurobehavioral competences, suggesting a disrupted neurological maturation. The results support the existence of forms of placental insufficiency not detected by current definitions of growth restriction."	
16	Triunfo 2017[20]	Yes	"All deliveries were attended by a staff obstetrician who was blinded to the results of the Doppler parameters evaluated."	No	Blinded	Negati ve	"In low risk pregnancies Doppler evaluation at 37 weeks of pregnancy did not improve the prediction of SGA and FGR by EFW, but combining EFW with Doppler boosted the prediction of APO by these parameters alone, although not crucially."	
17	Miranda 2017[21]	Unclear	"The laboratory personnel were blinded to the clinical	Yes	"In line with our clinical protocol, FGR cases were delivered	Not applica	"A multivariable integrative model at 32-36 weeks of	

			results or the outcomes of the patients."		electively at 37–38 weeks, while SGA cases were delivered at 40 weeks. The remaining pregnancies were allowed to continue, but elective delivery was offered at 41 weeks. SGA was defined as BW <10th centile. Fetuses with a BW of <10th centile and, additionally, suspected EFWc <10th centile and either abnormal CPR (<5th centile) or UtA-PI (≥95th centile) and/or a BW of <3rd centile according to local standards, were classified as FGR."	ble / Neutral	gestation modestly improved the detection of SGA and FGR as compared to EFW alone."	

Appendix S4c. References of accompanying studies to datasets included in the IPD.

1. Khalil AA, Morales-Rosello J, Morlando M, et al. Is fetal cerebroplacental ratio an independent predictor of intrapartum fetal compromise and neonatal unit admission? Am J Obstet Gynecol 2015; 213(1): 54 e1-10.
2. Khalil A, Morales-Rosello J, Townsend R, et al. Value of third-trimester cerebroplacental ratio and uterine artery Doppler indices as predictors of stillbirth and perinatal loss. Ultrasound Obstet Gynecol 2016; 47(1): 74-80.
3. D'Antonio F, Patel D, Chandrasekharan N, Thilaganathan B, Bhide A. Role of cerebroplacental ratio for fetal assessment in prolonged pregnancy. Ultrasound Obstet Gynecol 2013; 42(2): 196-200.
4. Vázquez-Sarandeses A, Gómez-Montes E, Herraiz I, Gómez-Arriaga PI, Quezada MS, Galindo A. Validation of a predictive risk model for adverse perinatal outcome in late-onset small for gestational age fetuses. J Matern Fetal Neonatal Med 2016; 29(S1): 17.
5. Severi FM, Bocchi C, Visentin A, et al. Uterine and fetal cerebral Doppler predict the outcome of third-trimester small-for-gestational age fetuses with normal umbilical artery Doppler. Ultrasound Obstet Gynecol 2002; 19(3): 225-8.
6. Vergani P, Roncaglia N, Ghidini A, et al. Can adverse neonatal outcome be predicted in late preterm or term fetal growth restriction? Ultrasound Obstet Gynecol 2010; 36(2): 166-70.
7. Karlsen HO, Ebbing C, Rasmussen S, Kiserud T, Johnsen SL. Use of conditional centiles of middle cerebral artery pulsatility index and cerebroplacental ratio in the prediction of adverse perinatal outcomes. Acta Obstet Gynecol Scand 2016; 95(6): 690-6.
8. Crimmins S, Rosenthal A, Kopelman J, Harman C, Turan O. Use of fetal Doppler in the prediction of outcome in the fetus that is appropriate for gestational age. Am J Obstet Gynecol 2016; 214(1): S173-S4.
9. Shannon A, Crimmins S, Kopelman J, Harman C, Turan O. Abnormal MCA dopplers in diabetic patients and the association with stillbirth. Reprod Sci 2017; 24(1): 250A.
10. Manogura AC, Turan O, Kush ML, et al. Predictors of necrotizing enterocolitis in preterm growth-restricted neonates. Am J Obstet Gynecol 2008; 198(6): 638.e1-5.
11. Jain S, Kasdaglis T, Sharoky C, et al. Cerebrouterine Doppler ratio (CUR) predicts adverse perinatal outcome near term. Am J Obstet Gynecol 2011; 204(1): S159-S60.
12. Bligh LN, Alsolai AA, Greer RM, Kumar S. Cerebroplacental ratio thresholds measured within 2 weeks before birth and risk of Cesarean section for intrapartum fetal compromise and adverse neonatal outcome. Ultrasound Obstet Gynecol 2018; 52(3): 340-6.
13. Bligh LN, Alsolai A, Greer RG, Kumar S. Prediction of intrapartum fetal compromise at term using the cerebro-umbilical ratio. J Pediatr 2016; 52(S2): 113-4.
14. Flatley C, Greer RM, Kumar S. Magnitude of change in fetal cerebroplacental ratio in third trimester and risk of adverse pregnancy outcome. Ultrasound Obstet Gynecol 2017; 50(4): 514-9.
15. Gibbons A, Flatley C, Kumar S. Cerebroplacental ratio in pregnancies complicated by gestational diabetes mellitus. Ultrasound Obstet Gynecol 2017; 50(2): 200-6.
16. Twomey S, Flatley C, Kumar S. The association between a low cerebro-umbilical ratio at 30-34 weeks gestation, increased intrapartum operative intervention and adverse perinatal outcomes. European journal of obstetrics, gynecology, and reproductive biology 2016; 203: 89-93.
17. Unterscheider J, Daly S, Geary MP, et al. Predictable progressive Doppler deterioration in IUGR: does it really exist? Am J Obstet Gynecol 2013; 209(6): 539.e1-7.
18. Flood K, Unterscheider J, Daly S, et al. The role of brain sparing in the prediction of adverse outcomes in intrauterine growth restriction: results of the multicenter PORTO Study. Am J Obstet Gynecol 2014; 211(3): 288.e1-5.
19. Mula R, Savchev S, Parra M, et al. Increased fetal brain perfusion and neonatal neurobehavioral performance in normally grown fetuses. Fetal diagnosis and therapy 2013; 33(3): 182-8.
20. Triunfo S, Crispi F, Gratacos E, Figueras F. Prediction of delivery of small-for-gestational-age neonates

and adverse perinatal outcome by fetoplacental Doppler at 37 weeks' gestation. Ultrasound Obstet Gynecol 2017; 49(3): 364-71.
21. Miranda J, Rodriguez-Lopez M, Triunfo S, et al. Prediction of fetal growth restriction using estimated fetal weight vs a combined screening model in the third trimester. Ultrasound Obstet Gynecol 2017; 50(5): 603-11.

Appendix S5. Results of QUADAS-2 assessment of risk of bias and applicability concerns.

**Dataset**	**RISK OF BIAS**				**APPLICABILITY CONCERNS**		
	PATIENT SELECTION	INDEX TEST	REFERENCE STANDARD	FLOW AND TIMING	PATIENT SELECTION	INDEX TEST	REFERENCE STANDARD
1	☺	☺	☹	☺	☺	☺	☺
2	☺	☺	☹	☺	☺	☺	☺
3	☺	?	?	☺	☺	☺	☺
4	☺	☹	?	☺	☺	☺	☺
5	☺	☺	☹	☺	☺	☺	☺
6	☺	☺	☹	☺	☺	☺	☺
7	☺	?	?	☺	☺	☺	☺
8	☺	☺	?	☺	☺	☺	☺
9	☺	☹	?	☺	☺	☺	☺
10	☺	☺	☺	☺	☺	☺	☺
11	☺	?	?	☺	☺	☺	☺
12	☺	☹	?	☺	☺	☺	☺
13	☺	☺	?	☺	☺	☺	☺
14	☺	☺	☹	☺	☺	☺	☺
15	☺	☺	☺	☺	☺	☺	☺
16	☺	☺	☺	☺	☺	☺	☺
17	☹	☹	?	☺	☺	☺	☺

☺ = low risk; ☹ = high risk; ? = unclear risk.

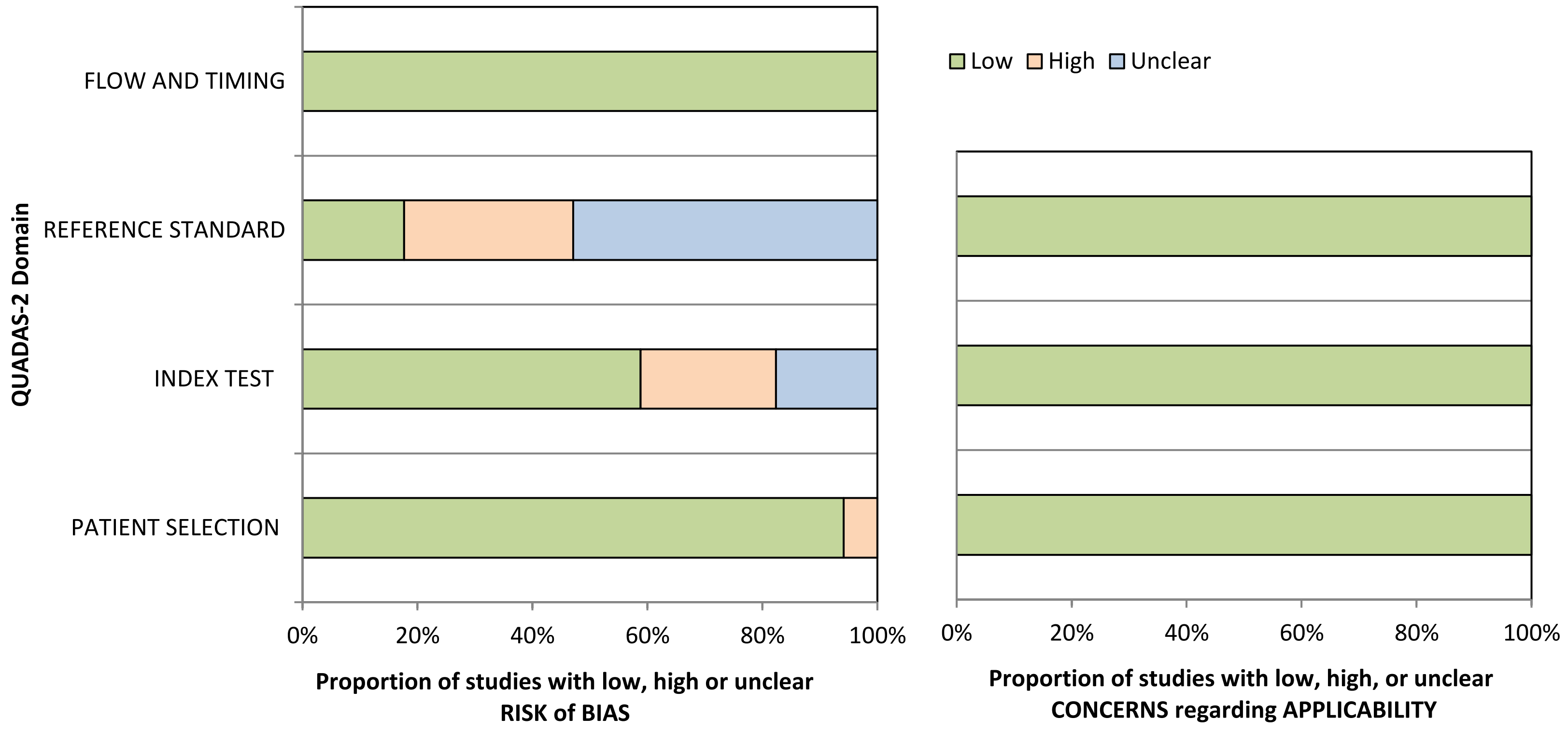
Low
High
Unclear
FLOW AND TIMING
REFERENCE STANDARD
INDEX TEST
PATIENT SELECTION
QUADAS-2 Domain
0%
20%
40%
60%
80%
100%
Proportion of studies with low, high or unclear RISK of BIAS
Proportion of studies with low, high, or unclear CONCERNS regarding APPLICABILITY

Appendix S6. Details of outcome measures reported in studies that contributed to the IPD.

Outcomes	Number of studies	Number of women	Mean (SD) or n (%)*
Time-to-delivery (days)	13	17 377	11.9 (14.8)
Gestational age at delivery (weeks)	17	21 632	39.3 (2.5)
Preterm delivery (< 37 weeks)	17	21 632	1369 (8.9%)
Preterm delivery (< 34 weeks)	17	21 632	836 (3.8%)
Birth weight (grams)	16	19 759	3152.2 (727.9)
Birth weight (centiles)	16	19 716	47.1 (32.3)
Birth weight < p10	16	19 716	3594 (18.2%)
Birth weight < p2.3	16	19 716	1213 (6.2%)
Mode of delivery			
Vaginal delivery	14	20 670	12 504 (60.5%)
Elective caesarean	14	20 670	1936 (9.4%)
Emergency caesarean	14	20 670	3024 (14.6%)
Instrumental	14	20 670	2337 (11.4%)
Perinatal death	14	20 040	121 (0.6%)
Stillbirth	14	20 040	35 (0.2%)
Neonatal death	14	20 040	79 (0.4%)
Emergency caesarean section for fetal distress	16	21 373	1696 (7.9%)
Apgar score at 5 minutes	12	7936	9.3 (0.9)
Apgar score <7 at 5 minutes	13	9019	145 (1.6%)
Arterial pH	12	5747	7.24 (0.08)
Acidosis**	13	8424	145 (1.7%)
Neonatal admission	14	19 420	2378 (12.2%)
Admission to NICU	9	6216	1150 (18.5%)
Admission to NCCU	3	4006	872 (21.8%)
Admission to NNU	1	9198	356 (3.9%)
Composite adverse outcome	12	18 731	3423 (18.3%)

*Percentage of number of cases

**Arterial cord bood pH <7.0 and/or base excess <-12mmol/L

Appendix S7. Areas-under-the-curves, in each included individual study, of UA PI, MCA PI, CPR, and CPR added to UA PI for the composite adverse perinatal outcome. Datasets 4, 10, and 15-17 were not included here, as the composite adverse outcome was not provided.

Dataset #	Proportion with adverse outcome (%)	AUC (95% CI)			
		UA PI	MCA PI	CPR	UA PI+CPR
1. Khalil 2015	1012 (11%)	0.512 (0.493-0.532)	0.563 (0.545-0.581)	0.563 (0.544-0.582)	0.509 (0.490-0.528)
2. D'Antonio 2013	61 (19%)	0.575 (0.496-0.655)	0.511 (0.436-0.586)	0.554 (0.475-0.634)	0.573 (0.494-0.653)
3. Vazquez 2016	39 (20%)	0.790 (0.699-0.881)	0.710 (0.630-0.790)	0.820 (0.742-0.897)	0.787 (0.695-0.879)
5. Vergani 2010	130 (28%)	0.734 (0.683-0.785)	0.533 (0.473-0.593)	0.687 (0.632-0.742)	0.733 (0.682-0.785)
6. Karlsen 2016	57 (28%)	0.809 (0.731-0.888)	0.633 (0.548-0.718)	0.784 (0.703-0.865)	0.810 (0.731-0.888)
7. Crimmins 2016	206 (20%)	0.493 (0.448-0.538)	0.536 (0.491-0.580)	0.522 (0.477-0.568)	0.492 (0.447-0.537)
8. Manogura 2008	389 (96%)	0.765 (0.645-0.885)	0.517 (0.323-0.711)	0.705 (0.571-0.838)	0.766 (0.646-0.885)
9. Jain 2011	277 (13%)	0.587 (0.549-0.626)	0.516 (0.479-0.554)	0.565 (0.527-0.604)	0.588 (0.550-0.626)
11. Flatley 2016	0	NA	NA	NA	NA
12. Gibbons 2017	218 (20%)	0.604 (0.559-0.649)	0.544 (0.500-0.587)	0.597 (0.551-0.642)	0.603 (0.558-0.647)
13. Twomey 2016	738 (60%)	0.611 (0.579-0.642)	0.606 (0.574-0.637)	0.640 (0.609-0.670)	0.608 (0.577-0.639)
14. Unterscheider 2013	296 (34%)	0.716 (0.678-0.753)	0.591 (0.551-0.631)	0.722 (0.685-0.760)	0.713 (0.675-0.751)

Appendix S8. Scatter plots.

Figure 1. Scatter plot of cases with adverse outcome (green dots; n=3423) and cases without adverse outcome (blue dots; n=15 308) with their respective UA PI values (x-axis, logarithmic) and CPR values (y-axis, logarithmic).

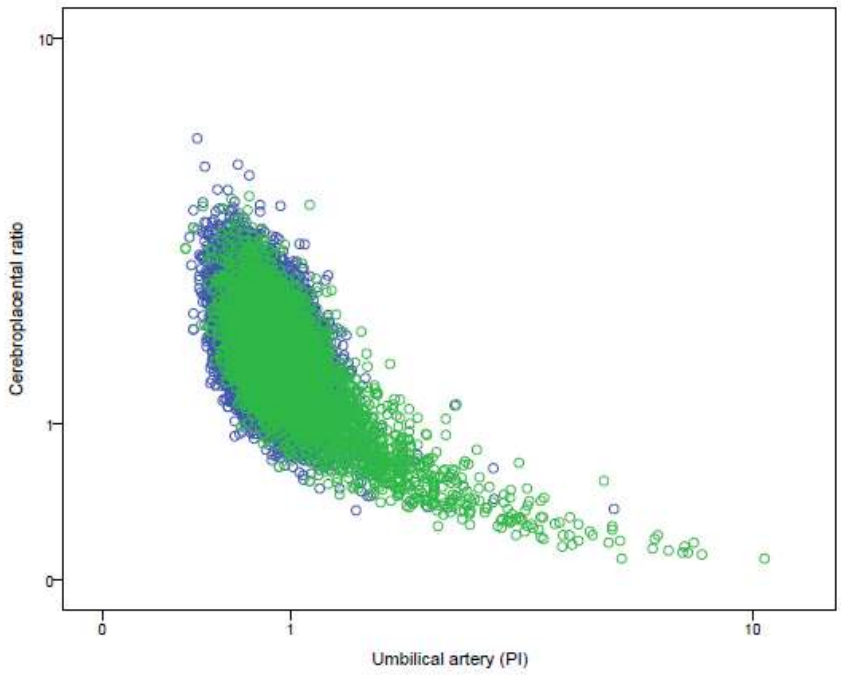

Figure 2. Scatter plot of cases with adverse outcome (green dots; n=3,423) and cases without adverse outcome (blue dots; n=15,308) with their respective middle cerebral artery pulsatility index values (x-axis, logarithmic) and cerebroplacental ratio values (y-axis, logarithmic).

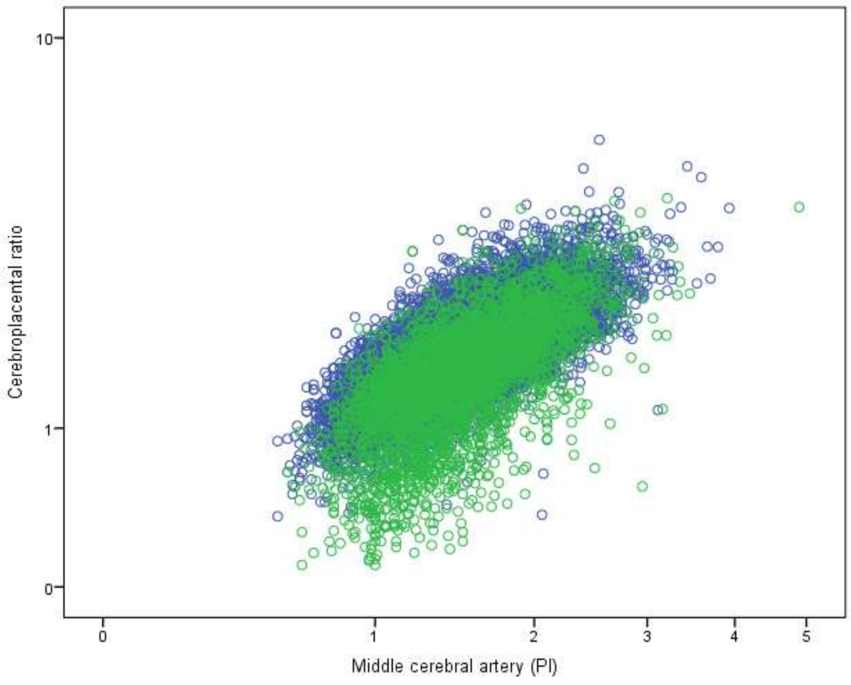

Figure 3. Scatter plot of cases with adverse outcome (green dots; n=3,423) and cases without adverse outcome (blue dots; n=15,308) with their respective umbilical artery pulsatility index values (x-axis, logarithmic) and middle cerebral artery pulsatility index values (y-axis, logarithmic).

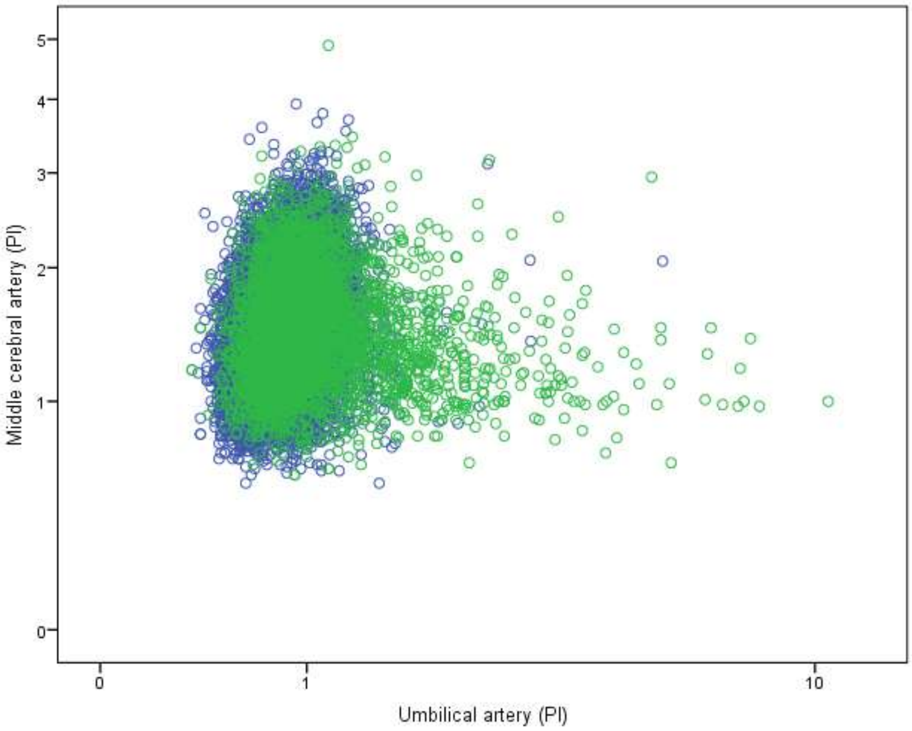

Appendix S9. Subgroup analyses presented in tables.

Table 1. Prediction of composite adverse perinatal outcome in subgroups according to gestational age at delivery

Subgroup	Model	OR	95% CI of OR	P-value of fit*	AUC	95% CI of AUC
GA <34 weeks n=785	UA PI	2.236	0.781-6.402	-	0.957	0.850-0.988
	MCA PI	0.340	0.105-1.104	-	0.959	0.863-0.989
	CPR	0.534	0.223-1.278	-	0.956	0.853-0.988
	UA PI + CPR	-	-	0.803	0.957	0.852-0.988
GA 34-37 weeks n=978	UA PI	5.365	2.876-10.007	-	0.778	0.657-0.864
	MCA PI	0.590	0.419-0.829	-	0.760	0.638-0.851
	CPR	0.497	0.386-0.639	-	0.773	0.653-0.860
	UA PI + CPR	-	-	0.035	0.780	0.660-0.866
GA ≥37 weeks n=15 297	UA PI	2.369	1.833-3.062	-	0.647	0.571-0.716
	MCA PI	0.638	0.544-0.748	-	0.656	0.581-0.723
	CPR	0.655	0.585-0.733	-	0.657	0.583-0.725
	UA PI + CPR	-	-	<0.001	0.656	0.581-0.724

Table 2. Prediction of composite adverse perinatal outcome in subgroups according to birth weight centile

Subgroup	Model	OR	95% CI of OR	P-value of fit*	AUC	95% CI of AUC
BW centile <10 n=3190	UA PI	9.334	6.391-13.630	-	0.836	0.760-0.891
	MCA PI	0.449	0.349-0.576	-	0.818	0.738-0.878
	CPR	0.290	0.234-0.359	-	0.836	0.760-0.891
	UA PI + CPR	-	-	<0.001	0.840	0.765-0.894
BW centile 10-25 n=2487	UA PI	7.594	4.543-12.693	-	0.786	0.680-0.865
	MCA PI	0.498	0.356-0.697	-	0.782	0.677-0.860
	CPR	0.423	0.331-0.541	-	0.790	0.685-0.866
	UA PI + CPR	-	-	0.004	0.790	0.685-0.867
BW centile ≥25 n=11 206	UA PI	2.678	1.954-3.671	-	0.763	0.667-0.839
	MCA PI	0.701	0.594-0.827	-	0.769	0.675-0.843
	CPR	0.702	0.623-0.791	-	0.770	0.676-0.844
	UA PI + CPR	-	-	0.002	0.768	0.673-0.842

Table 3. Prediction of composite adverse perinatal outcome in subgroups according to estimated fetal weight (EFW) centile in Complete Cases

Subgroup	Model	OR	95% CI of OR	P-value of fit*	AUC	95% CI of AUC
EFW centile <10 n=589	UA PI	18.508	9.155-37.418	-	0.776	0.738-0.813
	MCA PI	0.512	0.331-0.792	-	0.680	0.637-0.723
	CPR	0.204	0.139-0.299	-	0.765	0.726-0.803
	UA PI + CPR	-	-	0.007	0.781	0.744-0.818
EFW centile ≥10 n=3995	UA PI	3.348	2.321-4.828	-	0.598	0.733-0.623
	MCA PI	0.773	0.621-0.963	-	0.573	0.548-0.598
	CPR	0.676	0.574-0.795	-	0.589	0.564-0.614
	UA PI + CPR	-	-	0.258	0.598	0.573-0.622

*Multivariate analysis, p-value of adding CPR to model UA PI alone.
Abbreviations: BW = birth weight, UA = umbilical artery, MCA = middle cerebral artery, CPR = cerebroplacental ratio, PI = pulsatility index, OR = odds ratio, CI = confidence interval, AUC = area under the curve.
Composite outcome = perinatal death, emergency cesarean section for fetal distress, and neonatal admission

Appendix S10: Sensitivity analyses**.**

Table 1. Prediction of separate outcomes

Model	OR	95% CI of OR	P-value of fit*	AUC	95% CI of AUC
Perinatal death					
UA PI	1.807	1.477-2.213	-	0.855	0.817-0.893
MCA PI	0.751	0.472-1.196	-	0.835	0.795-0.876
CPR	0.403	0.284-0.571	-	0.841	0.799-0.883
UA PI + CPR	-	-	<0.001	0.846	0.805-0.888
Stillbirth					
UA PI	1.878	1.171-3.014	-	0.728	0.653-0.802
MCA PI	1.456	0.631-3.358	-	0.698	0.614-0.781
CPR	0.630	0.332-1.193	-	0.682	0.594-0.770
UA PI + CPR	-	-	0.470	0.711	0.635-0.788
Emergency cesarean section for fetal distress					
UA PI	1.711	1.497-1.956	-	0.667	0.654-0.680
MCA PI	0.415	0.356-0.484	-	0.687	0.674-0.699
CPR	0.468	0.420-0.520	-	0.690	0.677-0.703
UA PI + CPR	-	-	0.177	0.690	0.677-0.702
Neonatal admission					
UA PI	7.645	6.109-9.567	-	0.856	0.847-0.865
MCA PI	0.623	0.542-0.717	-	0.846	0.837-0.855
CPR	0.462	0.417-0.511	-	0.852	0.843-0.861
UA PI + CPR	-	-	<0.001	0.856	0.847-0.865
Apgar score <7 at 5 minutes					
UA PI	1.483	1.232-1.787	-	0.770	0.735-0.805
MCA PI	0.999	0.651-0.153	-	0.750	0.714-0.786
CPR	0.548	0.394-0.763	-	0.763	0.728-0.799
UA PI + CPR	-	-	0.007	0.770	0.735-0.805
*Acidosis***					
UA PI	1.516	1.189-1.934	-	0.822	0.795-0.850
MCA PI	0.984	0.627-1.548	-	0.815	0.788-0.843
CPR	0.799	0.582-1.095	-	0.820	0.794-0.847
UA PI + CPR	-	-	0.005	0.823	0.795-0.850

*Likelihood ratio test: comparison UA vs. UA+CPR.. **Arterial cord bood pH <7.0 and/or base excess <-12mmol/L

Figure 1. ROC curves of UA PI (black), MCA PI (red), CPR (orange) and UA PI and CPR combined (blue) for separate outcomes.

Perinatal death

UA PI | MCA PI | CPR | UA PI + CPR

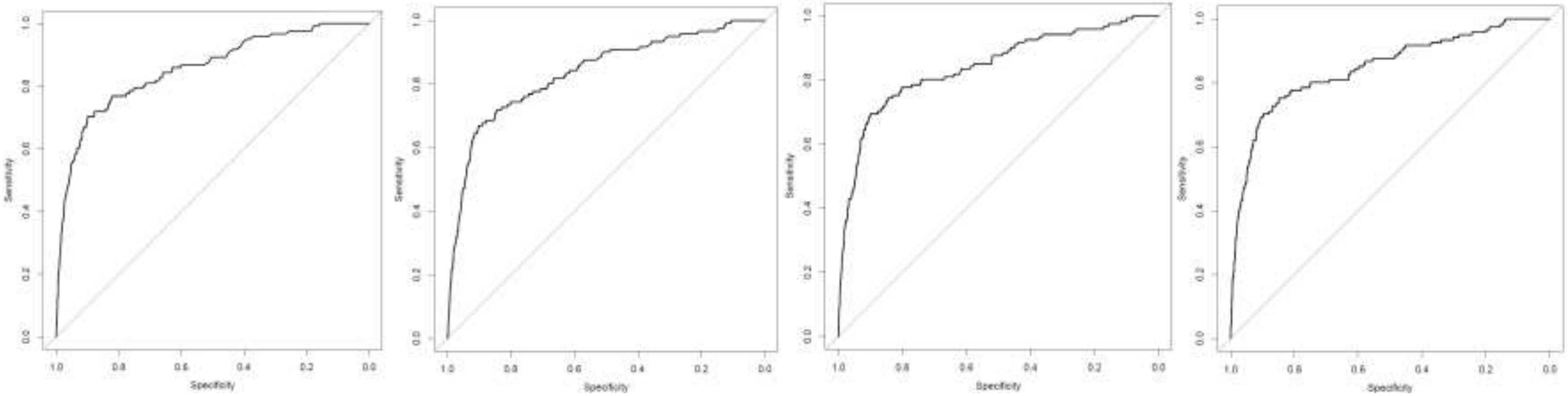

Stillbirth

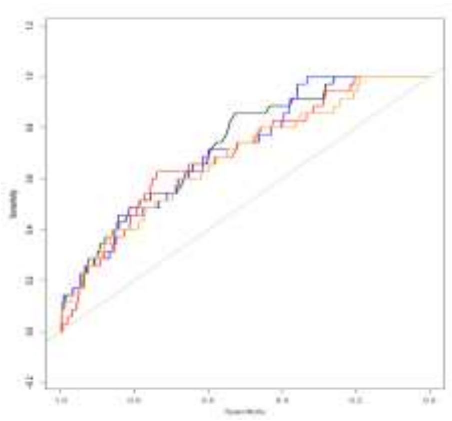

Emergency cesarean section for fetal distress

UA PI | MCA PI | CPR | UA PI + CPR

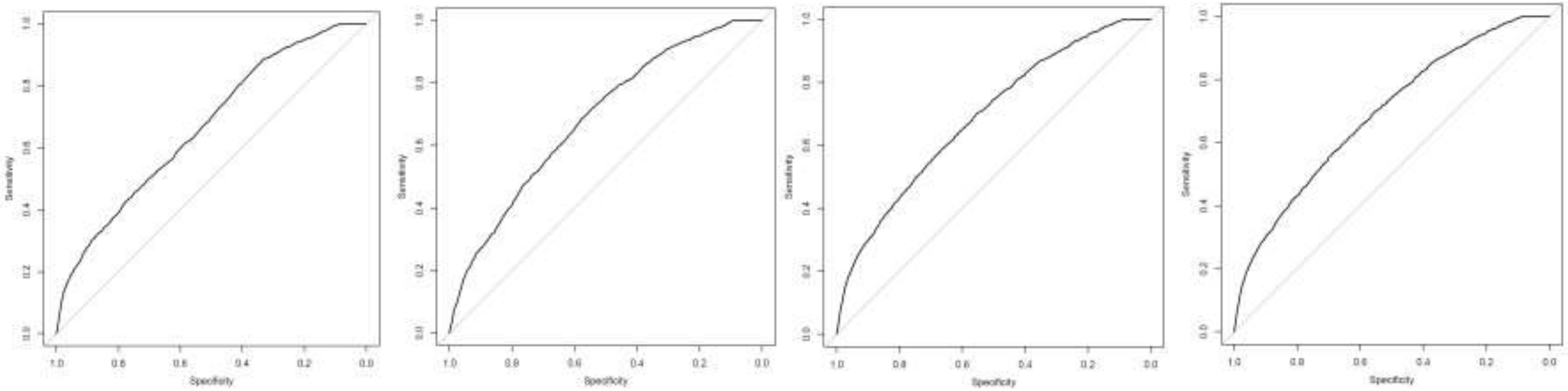

Neonatal admission

UA PI | MCA PI | CPR | UA PI + CPR

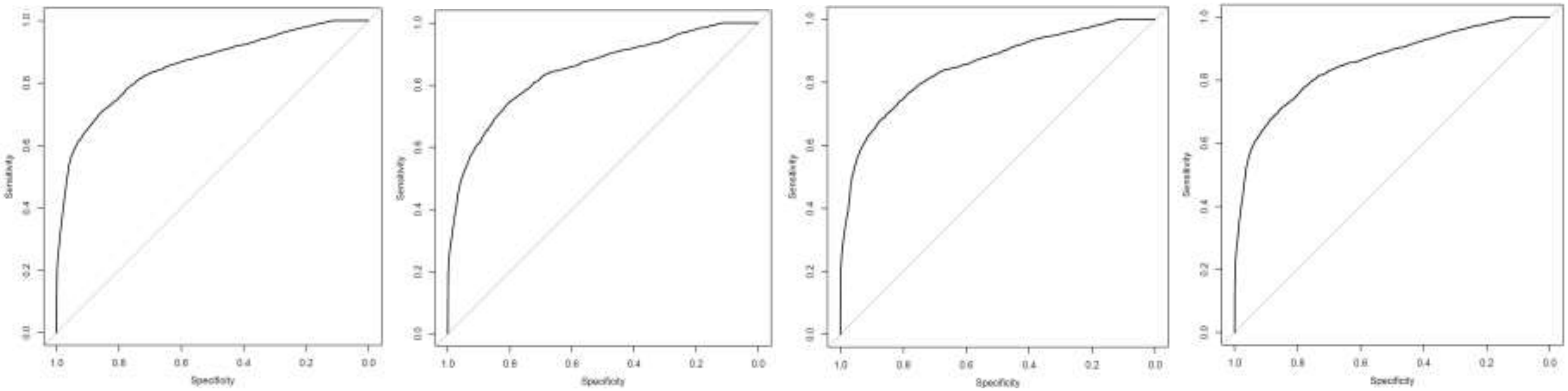

Apgar score <7 at 5 minutes

UA PI | MCA PI | CPR | UA PI + CPR

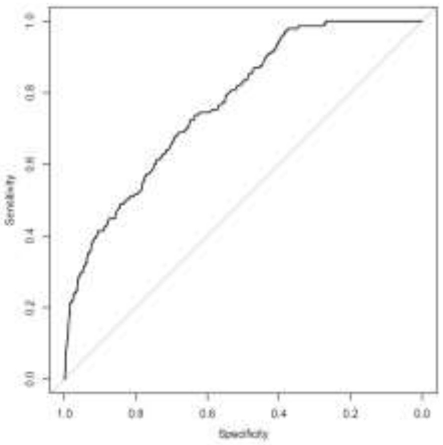

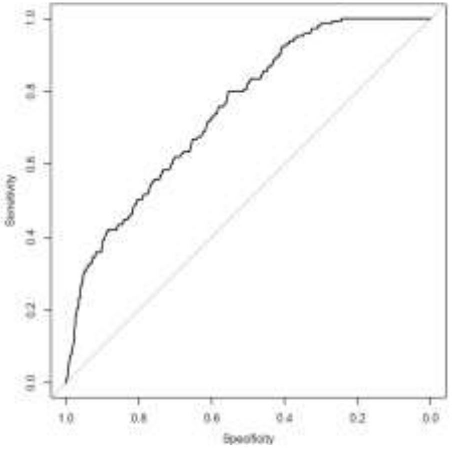

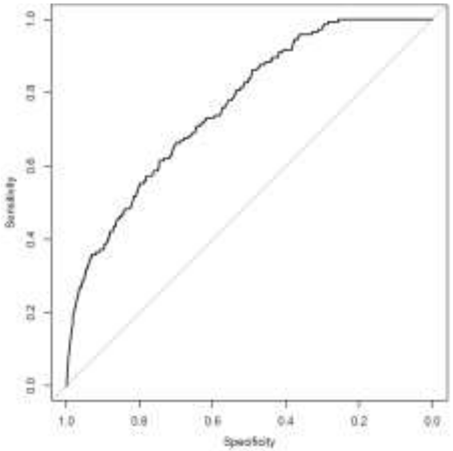

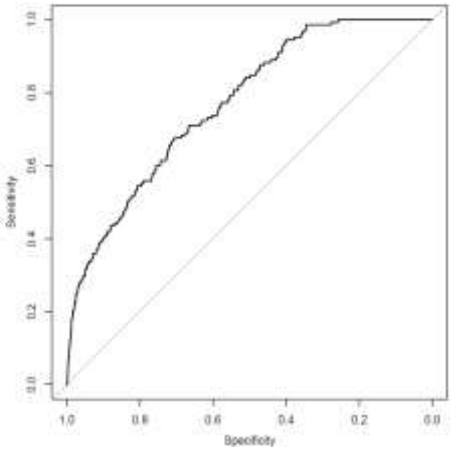

Acidosis

UA PI

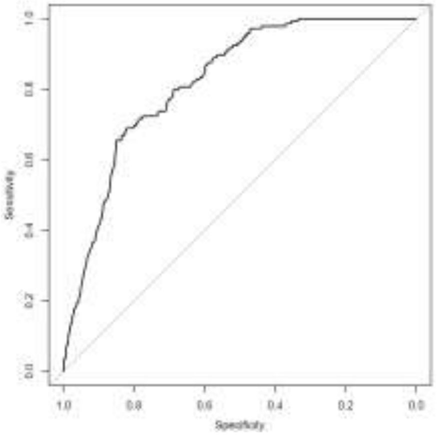

MCA PI

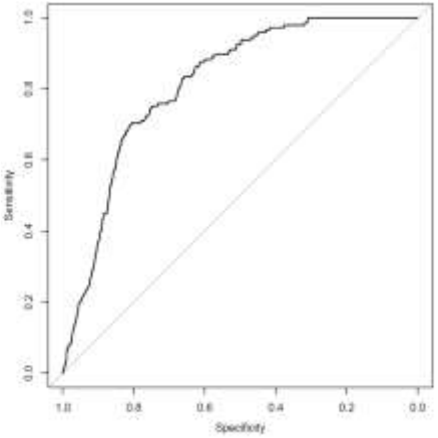

CPR

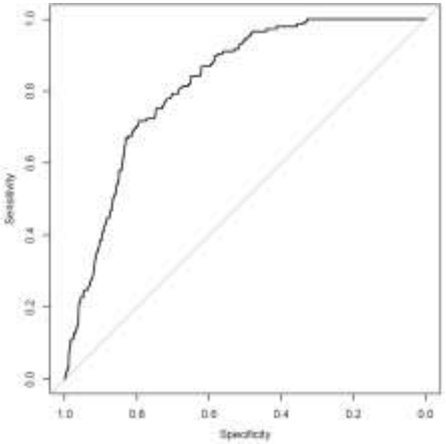

UA PI + CPR

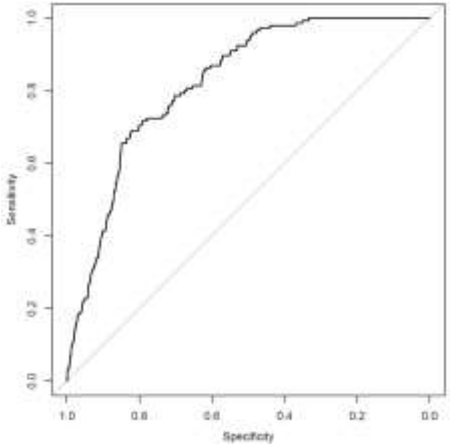

Table 2. Time between test and delivery

Test	Covariate	P-value of covariate
CPR	Time to delivery (days)	0.555

Table 3. Prediction of composite adverse perinatal outcome in patients with and without maternal hypertension

Model	OR	95% CI of OR	P-value of fit*	AUC	95% CI of AUC
Maternal hypertension (n=627)					
UA PI	19.965	1.529-52.941	-	0.865	0.837-0.893
MCA PI	0.321	0.194-0.533	-	0.848	0.818-0.878
CPR	0.299	0.207-0.430	-	0.863	0.835-0.892
UA PI + CPR	-	-	<0.001	0.768	0.759-0.778
No maternal hypertension (n=5456)					
UA PI	5.363	3.651-7.876	-	0.847	0.835-0.859
MCA PI	0.659	0.535-0.813	-	0.835	0.823-0.848
CPR	0.584	0.504-0.677	-	0.843	0.831-0.856
UA PI + CPR	-	-	<0.001	0.847	0.835-0.859

*Likelihood ratio test: comparison UA vs. UA+CPR.

Table 4. Prediction of composite adverse perinatal outcome in patients with and without maternal diabetes

Model	OR	95% CI of OR	P-value of fit*	AUC	95% CI of AUC
Maternal diabetes (n=1798)					
UA PI	14.63	7.336-19.182	-	0.731	0.702-0.759
MCA PI	0.662	0.476-0.920	-	0.689	0.659-0.718
CPR	0.511	0.406-0.642	-	0.720	0.692-0.749
UA PI + CPR	-	-	<0.001	0.768	0.759-0.778
No diabetes (n=4649)					
UA PI	5.29	3.619-7.743	-	0.917	0.908-0.925
MCA PI	0.543	0.431-0.684	-	0.912	0.903-0.921
CPR	0.517	0.438-0.609	-	0.917	0.908-0.923
UA PI + CPR	-	-	<0.001	0.917	0.909-0.926

*Likelihood ratio test: comparison UA vs. UA+CPR.

Table 5. Main analyses in Complete Cases: Prediction of composite adverse perinatal outcome in all patients

Model	OR	95% CI of OR	P-value of fit*	AUC	95% CI of AUC
UA PI	5.591	4.607 – 6.785	-	0.763	0.754-0.773
MCA PI	0.539	0.478 – 0.608	-	0.765	0.755-0.774
CPR	0.486	0.446 – 0.529	-	0.769	0.760-0.778
UA PI + CPR	-	-	<0.001	0.768	0.759-0.777
UA PI + MCA PI	-	-	<0.001	0.771	0.761-0.780

*Multivariate analysis, p-value of adding CPR to model UA alone.

Table 6. Prediction of composite adverse perinatal outcome in subgroups according to gestational age at delivery in Complete Cases

Subgroup	Model	OR	95% PI of OR	P-value of fit*	AUC	95% CI of AUC
GA <34 weeks n=856	UA PI	1.434	0.553-3.715	-	0.942	0.906-0.977
	MCA PI	0.332	0.093-1.19	-	0.952	0.923-0.982
	CPR	0.682	0.273-1.702	-	0.943	0.911-0.975
	UA PI + CPR	-	-	0.722	0.942	0.908-0.978
GA 34-37 weeks n=1092	UA PI	5.343	2.873-9.935	-	0.761	0.729-0.794
	MCA PI	0.606	0.433-0.847	-	0.745	0.712-0.778
	CPR	0.510	0.399-0.653	-	0.758	0.725-0.790
	UA PI + CPR	-	-	0.025	0.763	0.731-0.796
GA ≥37 weeks n=19 704	UA PI	2.196	1.711-2.817	-	0.649	0.637-0.662
	MCA PI	0.609	0.528-0.704	-	0.663	0.651-0.675
	CPR	0.649	0.587-0.718	-	0.663	0.650-0.675
	UA PI + CPR	-	-	<0.001	0.662	0.649-0.674

*Multivariate analysis, p-value of adding CPR to model UA alone.

Table 7. Prediction of composite adverse perinatal outcome in subgroups according to birth weight (BW) centile in Complete Cases

Subgroup	Model	OR	95% CI of OR	P-value of fit*	AUC	95% CI of AUC
BW centile <10 n=3594	UA PI	9.400	6.603-1.338	-	0.824	0.808-0.840
	MCA PI	0.454	0.356-0.579	-	0.808	0.792-0.825
	CPR	0.284	0.234-0.345	-	0.826	0.810-0.842
	UA PI + CPR	-	-	<0.001	0.829	0.813-0.845
BW centile 10-25 n=2929	UA PI	8.196	4.937-13.606	-	0.739	0.711-0.767
	MCA PI	0.465	0.339-0.638	-	0.735	0.708-0.762
	CPR	0.404	0.319-0.512	-	0.744	0.717-0.771
	UA PI + CPR	-	-	0.001	0.745	0.717-0.772
BW centile ≥25 n=13 193	UA PI	2.486	1.868-3.308	-	0.668	0.653-0.684
	MCA PI	0.688	0.587-0.806	-	0.680	0.665-0.695
	CPR	0.710	0.636-0.793	-	0.680	0.665-0.695
	UA PI + CPR	-	-	0.001	0.676	0.660-0.691

*Multivariate analysis, p-value of adding CPR to model UA alone.

Table 8. Prediction of composite adverse perinatal outcome in all only those 1800 patients who had had an elective cesarean section (209 with adverse outcome, 1591 without adverse outcome)

Model	OR	95% CI of OR	P-value of fit*	AUC	95% CI of AUC
UA PI	9.375	4.867-18.060	-	0.810	0.777-0.843
MCA PI	0.510	0.341-0.763	-	0.797	0.764-0.829
CPR	0.383	0.281-0.523	-	0.807	0.775-0.840
UA PI + CPR	-	-	0.011	0.812	0.780-0.845

*Multivariate analysis, p-value of adding CPR to model UA alone.

Table 9. Birth weight centile <10 at gestational age of delivery ≥34 weeks

Model	OR	95% CI of OR	P-value of fit*	AUC	95% CI of AUC
UA PI	6.230	4.228-9.178	-	0.754	0.732-0.775
MCA PI	0.524	0.407-0.675	-	0.742	0.721-0.764
CPR	0.368	0.302-0.450	-	0.759	0.738-0.780
UA PI + CPR	-	-	<0.001	0.761	0.740-0.782

* Multivariate analysis, p-value of adding CPR to model UA PI alone.

Figure 2. Subgroup of cases with birth weight centile <10 at gestational age ≥34 weeks: ROC curves of UA (black), MCA (red), CPR (orange) and UA PI+CPR (blue) for composite adverse perinatal outcome.

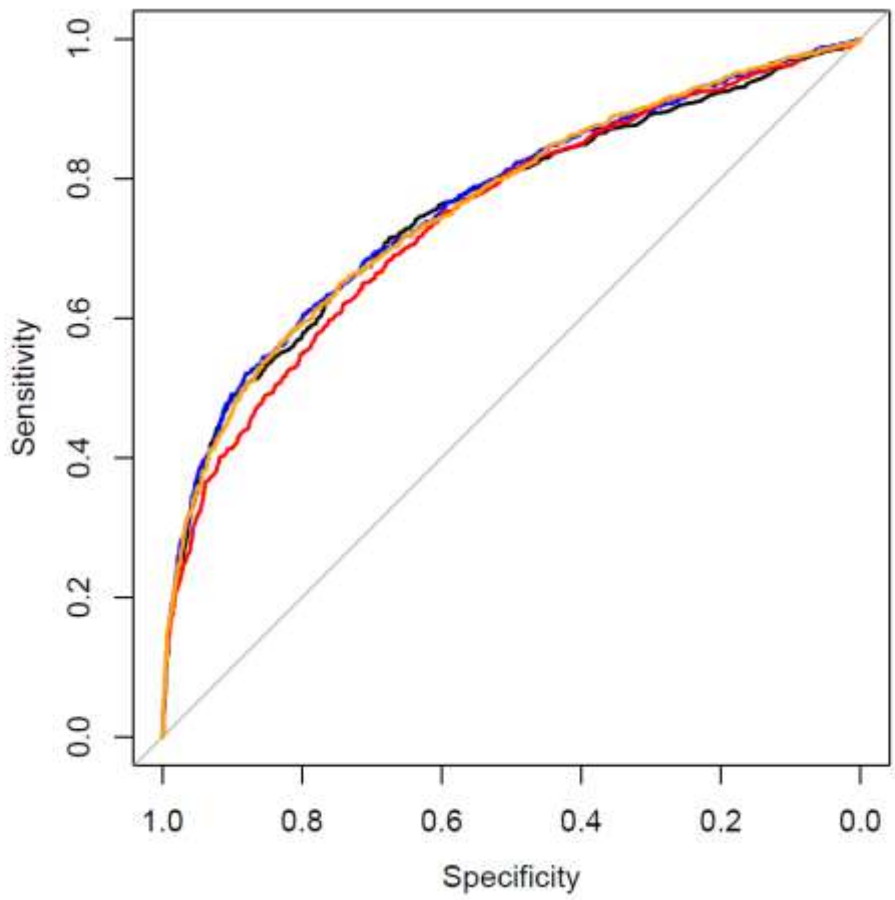

Appendix S11a. Studies not included in the IPD.

Study ID	Sample size	Reason	Test investigated	2x2 table extractable	Degree of conclusion positivity of studies on CPR
Akolekar 2015[1]	874	Unwilling since continuing area of researcher	CPR	Yes	Negative
Alatas 1996[2]	237	Without correct contact details	MCA	Yes	-
Bakalis 2015[3]	365	Unwilling since continuing area of researcher	CPR	Yes	Negative
Baschat 2007[4]	604	Data unavailable	MCA	Yes	-
Bayoumy 2005[5]	1037	Without correct contact details	CPR	Yes	Positive
Borowski 2000[6]	1730	Did not respond	CPR	No	Not applicable/neutral
Crimmins 2014[7]	987	Data unavailable	MCA	Yes	-
Cruz-Martinez 2011[8]	210	Data not provided within deadline	CPR and MCA	Yes	Positive
Dubiel 2002[9]	221	Did not respond	MCA	Yes	-
Figueras 2015[10]	509	Data not provided within deadline	CPR	Yes	Not applicable/neutral
Fong 1999[11]	293	Data unavailable	CPR and MCA	Yes	Positive
Gupta 2017[12]	200	Did not respond	CPR and MCA	Yes	Negative
Hajdo 2010[13]	216	Did not respond	MCA	No	-
Liu 2016[14]	476	Did not respond	CPR	No	Positive
Melamed 2016[15]	548	Data not published yet	CPR and MCA	Yes	Not applicable/neutral
Moga 2013[16]	350	Data unavailable	MCA	No	-
Nanthakomon 2010[17]	244	Did not respond	MCA	Yes	-
Ott 1999[18]	447	Without correct contact details	CPR	Yes	Positive
Ozyuncu 2010[19]	255	Did not respond	MCA	No	-
Pal 1991[20]	224	Without correct contact details	MCA	Yes	-
Prior 2015[21]	775	Data not provided within deadline	CPR	Yes	Negative
Qirko 2013[22]	318	Did not respond	CPR	No	Positive
Regan 2015[23]	270	Did not respond	CPR	Yes	Positive
Romero Gutiérrez 2009[24]	220	Did not respond	MCA	No	-
Sabdia 2015[25]	1381	Data not provided within deadline	CPR and MCA	Yes	Positive
Shahinaj 2010[26]	738	Did not respond	CPR	Yes	Positive

Shahinaj 2013[27]	283	Did not respond	Unclear	No	-
Sirico 2017[28]	3515	Legally unable to share	CPR	No	Negative
Stampalija 2017[29]	443	Requests of reserachers could not be met	CPR and MCA	No	Negative
Strigini 1997[30]	576	Did not respond	MCA	Yes	-
Subramanian 2016[31]	200	Did not respond	CPR and MCA	Yes	Positive
Thiebaugeorges 2006[32]	518	Data unavailable	MCA	Yes	-
Valino 2016[33]	3953	Unwilling since continuing area of researcher	MCA	Yes	-
Valino 2016[34]	8268	Unwilling since continuing area of researcher	MCA	Yes	-
Total	31 485				

Appendix S11b. References of studies not included in the IPD.

1. Akolekar R, Syngelaki A, Gallo DM, Poon LC, Nicolaides KH. Umbilical and fetal middle cerebral artery Doppler at 35-37 weeks' gestation in the prediction of adverse perinatal outcome. *Ultrasound Obstet Gynecol* 2015; **46**(1): 82-92.

2. Alatas C, Aksoy E, Akarsu C, Yakin K, Bahceci M. Prediction of perinatal outcome by middle cerebral artery Doppler velocimetry. *Arch Gynecol Obstet* 1996; **258**(3): 141-6.
3. Bakalis S, Akolekar R, Gallo DM, Poon LC, Nicolaides KH. Umbilical and fetal middle cerebral artery Doppler at 30-34 weeks' gestation in the prediction of adverse perinatal outcome. *Ultrasound Obstet Gynecol* 2015; **45**(4): 409-20.
4. Baschat AA, Cosmi E, Bilardo CM, et al. Predictors of neonatal outcome in early-onset placental dysfunction. *Obstet Gynecol* 2007; **109**(2 Pt 1): 253-61.
5. Bayoumy A, Crauciuc E, Pricop F. [Cerebro-umbilical ratio--high fidelity indicator in ante-partum fetal disorder]. *Rev Med Chir Soc Med Nat Iasi* 2005; **109**(3): 532-6.
6. Borowski D, Szaflik K, Kozarzewski M, et al. [Doppler evaluation as a predictor of asphyxia in fetuses with intrauterine growth retardation (IUGR)]. *Ginekol Pol* 2000; **71**(8): 828-32.
7. Crimmins S, Desai A, Block-Abraham D, Berg C, Gembruch U, Baschat AA. A comparison of Doppler and biophysical findings between liveborn and stillborn growth-restricted fetuses. *Am J Obstet Gynecol* 2014; **211**(6): 669.e1-10.
8. Cruz-Martinez R, Figueras F, Hernandez-Andrade E, Oros D, Gratacos E. Fetal brain Doppler to predict cesarean delivery for nonreassuring fetal status in term small-for-gestational-age fetuses. *Obstet Gynecol* 2011; **117**(3): 618-26.
9. Dubiel M, Gunnarsson GO, Gudmundsson S. Blood redistribution in the fetal brain during chronic hypoxia. *Ultrasound Obstet Gynecol* 2002; **20**(2): 117-21.
10. Figueras F, Savchev S, Triunfo S, Crovetto F, Gratacos E. An integrated model with classification criteria to predict small-for-gestational-age fetuses at risk of adverse perinatal outcome. *Ultrasound Obstet Gynecol* 2015; **45**(3): 279-85.
11. Fong KW, Ohlsson A, Hannah ME, et al. Prediction of perinatal outcome in fetuses suspected to have intrauterine growth restriction: Doppler US study of fetal cerebral, renal, and umbilical arteries. *Radiology* 1999; **213**(3): 681-9.
12. Gupta P, Mathur S, Khanna R. Doppler triple-vessel wave pattern as a screening method for prediction of perinatal outcome in pregnancy-induced hypertension. *Journal of SAFOG* 2017; **9**(2): 173-8.
13. Hajdo J, Wilczynski J, Szymczak W, Nowakowska D. [Evaluation of the relation between Doppler flow in maternal and fetal vessels and the risk of adverse neonatal outcome]. *Ginekol Pol* 2010; **81**(2): 99-104.
14. Liu J, Song G, Zhao G, Meng T. The Value of the Cerebroplacental Ratio for the Prediction of Intrapartum Fetal Monitoring in Low-Risk Term Pregnancies. *Gynecol Obstet Invest* 2017; **82**(5): 475-80.
15. Melamed N, Pittini A, Kingdom J, Barrett J. Sonographic factors distinguishing late intrauterine growth restriction from late small for gestational age fetuses. *American Journal of Obstetrics and Gynecology* 2016; **214**(1): S104-S5.
16. Moga M, Gliga F, Anastasiu C, Mihalache M. Doppler velocimetry screening at 35 weeks of gestational age in symmetrically growth restricted fetuses (type III carrera)-predictive indicators. *Journal of Perinatal Medicine* 2013; **41**.
17. Nanthakomon T, Uerpairojkit B. Outcome of small-for-gestational-age fetuses according to umbilical artery Doppler: is there any yield from additional middle cerebral artery Doppler? *J Matern Fetal Neonatal Med* 2010; **23**(8): 900-5.
18. Ott WJ. Comparison of the non-stress test with the evaluation of centralization of blood flow for the prediction of neonatal compromise. *Ultrasound Obstet Gynecol* 1999; **14**(1): 38-41.
19. Ozyuncu O, Saygan-Karamursel B, Armangil D, et al. Fetal arterial and venous Doppler in growth restricted fetuses for the prediction of perinatal complications. *Turk J Pediatr* 2010; **52**(4): 384-92.
20. Pal A, Ulrich G, Manfred H. [Prognostic value of the study of the blood flow in the fetal median cerebral artery]. *Orv Hetil* 1991; **132**(33): 1815-7.
21. Prior T, Paramasivam G, Bennett P, Kumar S. Are fetuses that fail to achieve their growth potential at increased risk of intrapartum compromise? *Ultrasound Obstet Gynecol* 2015; **46**(4): 460-4.

22. Qirko R, Beka V, Shpati D, Haxhihyseni A. Association of cerebro-placental ratio with the newborns outcomes in preeclampsia. *Journal of Perinatal Medicine* 2013; **41**.
23. Regan J, Masters H, Warshak CR. Association between an abnormal cerebroplacental ratio and the development of severe pre-eclampsia. *J Perinatol* 2015; **35**(5): 322-7.
24. Romero Gutierrez G, Ramirez Hernandez GL, Molina Rodriguez R, Ponce de Leon AL, Cortes Salim P. [Predictive value of Doppler fluxometry of umbilical and middle cerebral arteries with the perinatal outcomes in fetus with intrauterine growth restriction]. *Ginecol Obstet Mex* 2009; **77**(1): 19-25.
25. Sabdia S, Greer RM, Prior T, Kumar S. Predicting intrapartum fetal compromise using the fetal cerebro-umbilical ratio. *Placenta* 2015; **36**(5): 594-8.
26. Shahinaj R, Manoku N, Kroi E, Tasha I. The value of the middle cerebral to umbilical artery Doppler ratio in the prediction of neonatal outcome in patient with preeclampsia and gestational hypertension. *J Prenat Med* 2010; **4**(2): 17-21.
27. Shahinaj R, Tasha I, Manoku N. Doppler velocimetry in postterm pregnancies complicated by oligohydramnios. *Journal of Perinatal Medicine* 2013; **41**.
28. Sirico A, Diemert A, Glosemeyer P, Hecher K. Prediction of adverse perinatal outcome by cerebroplacental ratio adjusted for estimated fetal weight. *Ultrasound Obstet Gynecol* 2018; **51**(3): 381-6.
29. Stampalija T, Arabin B, Wolf H, Bilardo CM, Lees C. Is middle cerebral artery Doppler related to neonatal and 2-year infant outcome in early fetal growth restriction? *Am J Obstet Gynecol* 2017; **216**(5): 521.e1-.e13.
30. Strigini FA, De Luca G, Lencioni G, Scida P, Giusti G, Genazzani AR. Middle cerebral artery velocimetry: different clinical relevance depending on umbilical velocimetry. *Obstet Gynecol* 1997; **90**(6): 953-7.
31. Subramanian V, Venkat J, Dhanapal M. Which is Superior, Doppler Velocimetry or Non-stress Test or Both in Predicting the Perinatal Outcome of High-Risk Pregnancies. *J Obstet Gynaecol India* 2016; **66**(Suppl 1): 149-56.
32. Thiebaugeorges O, Ancel PY, Goffinet F, Breart G. A population-based study of 518 very preterm neonates from high-risk pregnancies: prognostic value of umbilical and cerebral artery Doppler velocimetry for mortality before discharge and severe neurological morbidity. *Eur J Obstet Gynecol Reprod Biol* 2006; **128**(1-2): 69-76.
33. Valino N, Giunta G, Gallo DM, Akolekar R, Nicolaides KH. Biophysical and biochemical markers at 35-37 weeks' gestation in the prediction of adverse perinatal outcome. *Ultrasound Obstet Gynecol* 2016; **47**(2): 203-9.
34. Valino N, Giunta G, Gallo DM, Akolekar R, Nicolaides KH. Biophysical and biochemical markers at 30-34 weeks' gestation in the prediction of adverse perinatal outcome. *Ultrasound Obstet Gynecol* 2016; **47**(2): 194-202.

Appendix S11c. Reported accuracy estimates of CPR in studies unavailable for the IPD versus in studies included in the IPD.

CPR – Perinatal death

Study	TP	FP	FN	TN	Inclusion in IPD	Sensitivity (95% CI)	Specificity (95% CI)
Flood 2014	3	143	0	735	Included in the IPD	1.00 [0.29, 1.00]	0.84 [0.81, 0.86]
Khalil 2016	6	265	4	2529	Included in the IPD	0.60 [0.26, 0.88]	0.91 [0.89, 0.92]
Lalthantluanga 2015	15	31	4	50	Included in the IPD	0.79 [0.54, 0.94]	0.62 [0.50, 0.72]
Twomey 2016	8	67	38	1111	Included in the IPD	0.17 [0.08, 0.31]	0.94 [0.93, 0.96]
Bakalis 2015	1	84	4	276	Unavailable for the IPD	0.20 [0.01, 0.72]	0.77 [0.72, 0.81]
Regan 2015	10	30	0	230	Unavailable for the IPD	1.00 [0.69, 1.00]	0.88 [0.84, 0.92]
Shahinaj 2010	97	217	1	423	Unavailable for the IPD	0.99 [0.94, 1.00]	0.66 [0.62, 0.70]

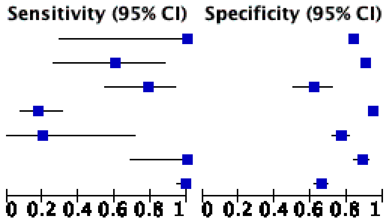

CPR – ED for fetal distress

Study	TP	FP	FN	TN	Inclusion in IPD	Sensitivity (95% CI)	Specificity (95% CI)
Karlsen 2016	29	18	21	137	Included in the IPD	0.58 [0.43, 0.72]	0.88 [0.82, 0.93]
Khalil 2015 – p54	188	649	1253	6292	Included in the IPD	0.13 [0.11, 0.15]	0.91 [0.90, 0.91]
Twomey 2016	25	50	107	1042	Included in the IPD	0.19 [0.13, 0.27]	0.95 [0.94, 0.97]
Akolekar 2015	8	52	50	521	Unavailable for the IPD	0.14 [0.06, 0.25]	0.91 [0.88, 0.93]
Bakalis 2015	8	16	19	144	Unavailable for the IPD	0.30 [0.14, 0.50]	0.90 [0.84, 0.94]
Cruz-Martinez 2011	28	32	33	117	Unavailable for the IPD	0.46 [0.33, 0.59]	0.79 [0.71, 0.85]
Ott 1999-UOG	11	36	27	373	Unavailable for the IPD	0.29 [0.15, 0.46]	0.91 [0.88, 0.94]
Prior 2015	18	31	82	644	Unavailable for the IPD	0.18 [0.11, 0.27]	0.95 [0.94, 0.97]
Regan 2015	24	16	30	200	Unavailable for the IPD	0.44 [0.31, 0.59]	0.93 [0.88, 0.96]
Sabdia 2015	31	110	93	1147	Unavailable for the IPD	0.25 [0.18, 0.34]	0.91 [0.90, 0.93]
Shahinaj 2010	187	73	265	159	Unavailable for the IPD	0.41 [0.37, 0.46]	0.69 [0.62, 0.74]

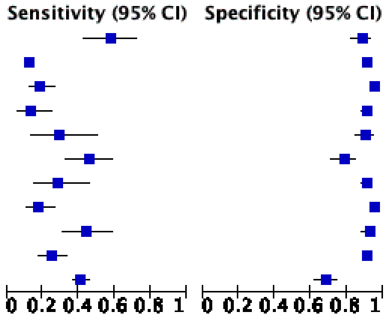

CPR – Apgar <7 at 5 minutes

Study	TP	FP	FN	TN	Inclusion in IPD	Sensitivity (95% CI)	Specificity (95% CI)
Lalthantluanga 2015	22	24	11	43	Included in the IPD	0.67 [0.48, 0.82]	0.64 [0.52, 0.76]
Akolekar 2015	2	78	5	687	Unavailable for the IPD	0.29 [0.04, 0.71]	0.90 [0.87, 0.92]
Bakalis 2015	3	51	3	219	Unavailable for the IPD	0.50 [0.12, 0.88]	0.81 [0.76, 0.86]
Bayoumy 2005	140	11	30	856	Unavailable for the IPD	0.82 [0.76, 0.88]	0.99 [0.98, 0.99]
Prior 2015	1	48	9	717	Unavailable for the IPD	0.10 [0.00, 0.45]	0.94 [0.92, 0.95]
Sabdia 2015	3	138	18	1219	Unavailable for the IPD	0.14 [0.03, 0.36]	0.90 [0.88, 0.91]
Shahinaj 2010	161	99	95	329	Unavailable for the IPD	0.63 [0.57, 0.69]	0.77 [0.73, 0.81]

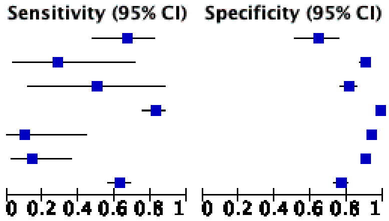

CPR – Acidosis at birth arterial

Study	TP	FP	FN	TN	Inclusion in IPD	Sensitivity (95% CI)	Specificity (95% CI)
Twomey 2016	8	37	89	386	Included in the IPD	0.08 [0.04, 0.16]	0.91 [0.88, 0.94]
Akolekar 2015	2	31	4	254	Unavailable for the IPD	0.33 [0.04, 0.78]	0.89 [0.85, 0.92]
Bakalis 2015	2	27	4	140	Unavailable for the IPD	0.33 [0.04, 0.78]	0.84 [0.77, 0.89]
Cruz-Martinez 2011	6	54	10	140	Unavailable for the IPD	0.38 [0.15, 0.65]	0.72 [0.65, 0.78]
Prior 2015	13	36	223	503	Unavailable for the IPD	0.06 [0.03, 0.09]	0.93 [0.91, 0.95]

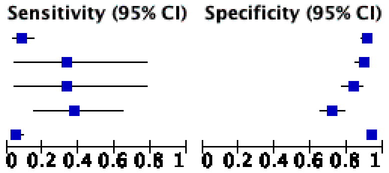

CPR – NICU admission

Study	TP	FP	FN	TN	Inclusion in IPD	Sensitivity (95% CI)	Specificity (95% CI)
Flood 2014	93	53	163	572	Included in the IPD	0.36 [0.30, 0.43]	0.92 [0.89, 0.94]
Karlsen 2016	32	15	15	143	Included in the IPD	0.68 [0.53, 0.81]	0.91 [0.85, 0.95]
Lalthantluanga 2015	26	20	15	39	Included in the IPD	0.63 [0.47, 0.78]	0.66 [0.53, 0.78]
Akolekar 2015	7	87	33	747	Unavailable for the IPD	0.17 [0.07, 0.33]	0.90 [0.87, 0.92]
Bakalis 2015	28	56	70	206	Unavailable for the IPD	0.29 [0.20, 0.39]	0.79 [0.73, 0.83]
Ott 1999-UOG	36	11	113	287	Unavailable for the IPD	0.24 [0.18, 0.32]	0.96 [0.93, 0.98]
Regan 2015	32	8	49	181	Unavailable for the IPD	0.40 [0.29, 0.51]	0.96 [0.92, 0.98]
Shahinaj 2010	202	58	201	223	Unavailable for the IPD	0.50 [0.45, 0.55]	0.79 [0.74, 0.84]

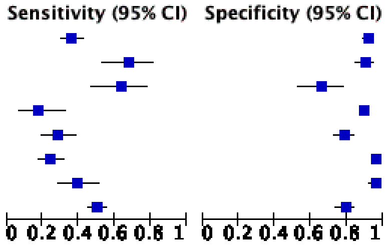

Supplement: Supplementary file 1 — Appendix S1. Search strategy. Appendix S2. Study selection. Appendix S3. Data changes. Appendix S4. (a) Characteristics of data sets included in the IPD. (b) Blinding of study results and conclusion scoring of data sets included in the IPD. (c) References of accompanying studies to data sets included in the IPD. Appendix S5. Results of QUADAS‐2 assessment of risk of bias and applicability concerns. Appendix S6. Details of outcome measures reported in studies that contributed to the IPD. Appendix S7. Areas‐under‐the‐curves, in each included individual study, of UA PI, MCA PI, CPR and CPR added to UA PI for the composite adverse perinatal outcome. Data sets 4, 10 and 15–17 were not included here, as the composite adverse outcome was not provided. Appendix S8. Scatter plots. Appendix S9. Subgroup analyses presented in tables. Appendix S10. Sensitivity analyses. Appendix S11. (a) Studies not included in the IPD. (b) References of studies not included in the IPD. (c) Reported accuracy estimates of CPR in studies unavailable for the IPD versus in studies included in the IPD. [file BJO-128-226-s001.zip › bjo16287-sup-0001-AppendixS1-S11.pdf]
